# Ocean currents and environmental gradients shape prokaryotic community structure and function in the South China Sea

**DOI:** 10.1128/spectrum.01020-25

**Published:** 2025-09-24

**Authors:** Yu Wang, Jinxin Xu, Yanting Liu, Lu Liu, Shicong Xiao, Xiaomeng Wang, Jiandong Zhang, Sijun Huang, Qiang Zheng

**Affiliations:** 1 State Key Laboratory for Marine Environmental Science, Institute of Marine Microbes and Ecospheres, College of Ocean and Earth Sciences, Xiamen University 534813, Xiamen, People’s Republic of China; 2 Fujian Key Laboratory of Marine Carbon Sequestration, Xiamen University 12466, Xiamen, People’s Republic of China; 3 College of the Environment and Ecology, Xiamen University 598522, Xiamen, People’s Republic of China; 4 Jiangsu Institute of Marine Resources Development, Jiangsu Ocean University 66486, Lianyungang, People’s Republic of China; 5 Department of Ocean Science and Engineering, Southern University of Science and Technology 255310, Shenzhen, People’s Republic of China; 6 CAS Key Laboratory of Tropical Marine Bio-resources and Ecology, South China Sea Institute of Oceanology, Chinese Academy of Sciences 74718 , Guangzhou, People’s Republic of China; National Center for Genetic, Khlong Luang, Pathum Thani, Thailand

**Keywords:** South China Sea, ocean currents, prokaryotic communities, community assembly mechanism, environmental selection, stochasticity

## Abstract

The South China Sea (SCS) is characterized by complex hydrodynamic conditions that influence the structure and function of prokaryotic microbial communities. This study conducted a comprehensive analysis of prokaryotic diversity, community assembly, and functional potential across various water masses within the SCS. Using 16S rRNA gene sequencing and co-occurrence network analyses, we found that geographic distance and environmental gradients, particularly temperature and nutrient levels, significantly impacted community composition. Our findings indicate that ecological drift is the primary mechanism governing community assembly, with spatial turnover primarily driven by the dispersal of microorganisms facilitated by ocean currents. Distinct modules in co-occurrence networks were associated with specific environmental factors, reflecting potential environmental selection processes along the SCS current. Keystone species and biomarkers identified through network analysis and random forest modeling exhibited varying associations with environmental variables, highlighting their adaptability to changing conditions. This work underscores the importance of ocean currents and environmental factors in shaping prokaryotic community dynamics and provides insights into microbial biogeography and ecosystem function in the SCS.

**IMPORTANCE:**

Microorganisms, especially prokaryotes, are fundamental in sustaining marine ecosystems through nutrient cycling and organic matter decomposition. However, understanding what shapes their diversity and distribution remains challenging. Our study highlights the significant role ocean currents and environmental conditions play in influencing prokaryotic communities in the South China Sea—a critical marine environment due to its dynamic currents and ecological complexity. We found that currents facilitate microbial dispersal, shaping community composition over vast areas, while temperature gradients act as key selective pressures, determining which species thrive. Additionally, we reveal that both predictable environmental selection and random ecological drift significantly contribute to community structuring. By identifying keystone microbes and biomarkers sensitive to environmental change, our work offers essential insights into marine microbial ecology. These findings are crucial for predicting how microbial communities, and thus ocean health and productivity, respond to ongoing environmental changes.

## INTRODUCTION

Prokaryotes play a paramount role in marine ecosystems as key drivers in the cycling of major elements such as carbon, nitrogen, and sulfur, thereby forming the foundation of marine microbial food webs (1–3). The structure and diversity of microbial communities not only reflect their ecological functions but also influence primary production, organic matter decomposition, and carbon sequestration (4). Keystone species are crucial components of microbial communities that exert a significant influence on community structure and function, regardless of their abundance (5). Additionally, biomarkers are specific indicators that can distinguish between different microbial communities, such as those in the plastisphere and the aquatic environment, and are identified using models like random-forest machine learning (6). In marine ecosystems, these keystone taxa and biomarkers can drive biogeochemical processes and maintain ecosystem stability (6–8). Identifying and understanding these taxa is vital for predicting ecosystem responses to environmental changes, as they can modulate the effects of disturbances and contribute to the resilience of marine ecosystems (6). Understanding the factors that shape these communities is essential for predicting how marine ecosystems respond to environmental changes (9).

Ocean currents play a crucial role in shaping the diversity and distribution of marine microorganisms by modulating nutrient availability and facilitating the dispersal of species across vast distances (10–12). Currents influence nutrient circulation and productivity, which, in turn, affect microbial metabolism and community composition (13). For instance, upwelling currents bring nutrient-rich deep waters to the surface, stimulating phytoplankton blooms that form the foundation of the marine food web (14). These physical processes have cascading effects on higher trophic levels, illustrating the interconnectedness of marine life (15). Recent research has revealed that ocean currents not only transport microorganisms but also create dynamic environmental gradients that drive microbial adaptation and evolution (16, 17). Microbial hitchhiking on particulate matter and debris carried by currents can lead to the colonization of new habitats, enhancing genetic diversity and promoting the spread of functional traits (18). Additionally, the interaction between ocean currents and mesoscale eddies generates unique microenvironments that can harbor distinct microbial communities, contributing to regional biodiversity hotspots (19).

The South China Sea (SCS), the largest tropical-subtropical marginal sea, presents a unique environment to study these phenomena (20). Covering approximately 3.5  ×  10^6^ km², it exhibits a variety of oceanographic processes akin to those in major ocean basins, including complex circulation patterns driven by monsoonal winds, mesoscale eddies, coastal upwelling, and significant riverine inputs (19). However, its semi-enclosed nature and smaller size relative to open oceans offer a more constrained system to investigate how physical processes influence microbial communities (21). In the SCS, the upper-layer circulation exhibits pronounced seasonal characteristics, primarily driven by monsoons (20). This circulation not only facilitates the dispersal of marine microorganisms but also influences nutrient influx and distribution (22). Prolonged stratification due to high temperatures can hinder nutrient replenishment from deeper layers, affecting microbial productivity (23). Mesoscale eddies are prevalent and can modulate nutrient conditions by displacing isopycnals within the euphotic zone, impacting microbial distribution and activity (24, 25). Coastal upwelling brings nutrient-rich, cold water to the surface, enhancing primary productivity and altering microbial community structures (26). Additionally, riverine inputs modify microbial community composition by introducing nutrients and organic matter, influencing microbial metabolic capacities (27).

Despite advances in regional studies (28–30), comprehensive basin-wide analyses of microbial diversity and distribution in the SCS remain limited. The dynamic environment of the SCS provides an opportunity to examine how large-scale oceanic processes influence microbial communities on a regional scale. Here, we present a broad survey of the SCS region to (i) analyze the spatial distribution patterns of prokaryotic microbial communities and diversity in the South China Sea, (ii) elucidate the mechanisms underlying the assembly of prokaryotic microbial communities in the SCS, and (iii) explore the influence of oceanic currents on the distribution of prokaryotic microorganisms, particularly the keystone species. By investigating these aspects, we aim to enhance the understanding of how ocean currents shape microbial community structure and function in the SCS, with implications for biogeochemical cycling and ecosystem health.

## MATERIALS AND METHODS

### Study area and sampling

**Fig 1:**
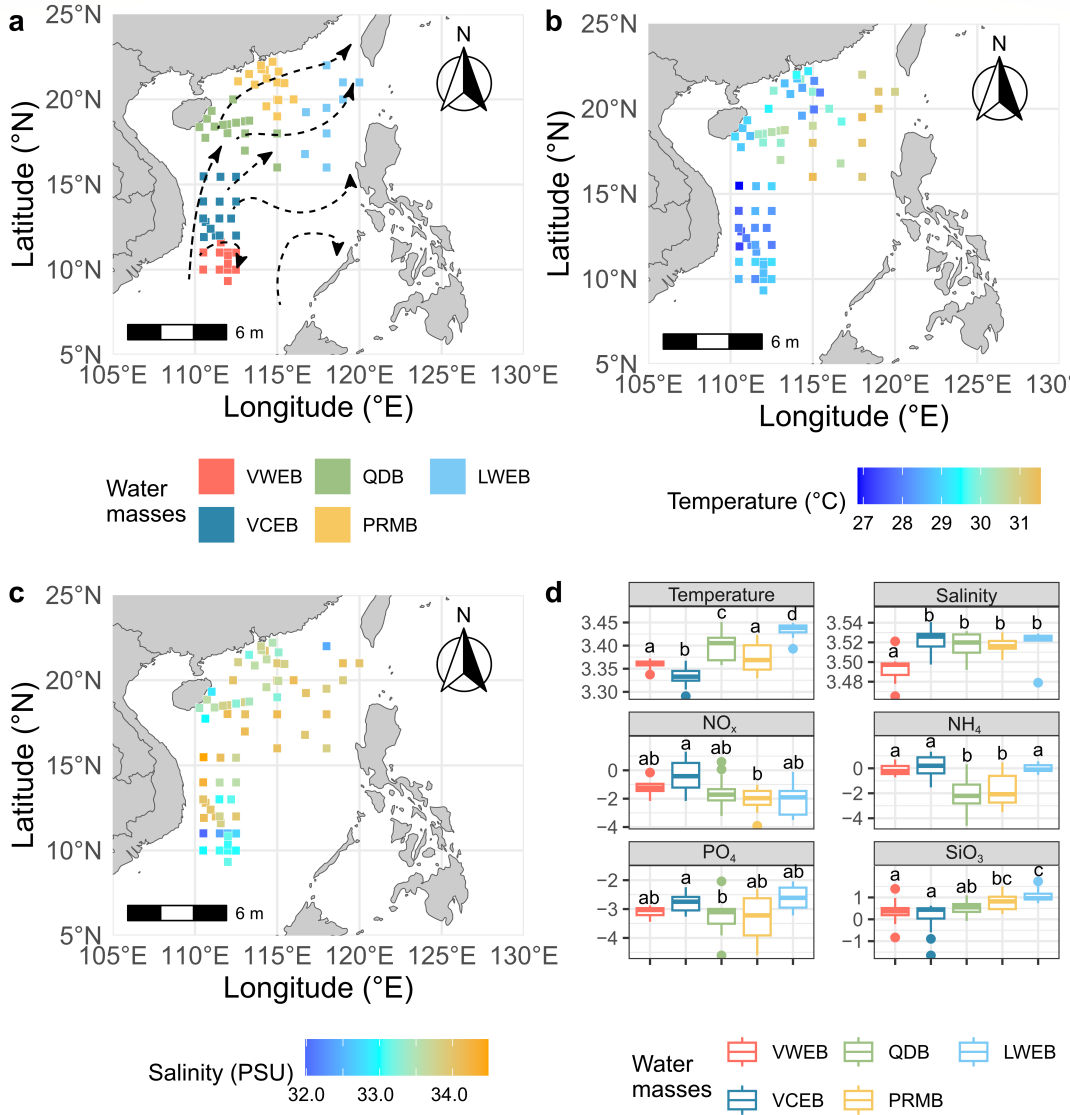
Water masses and environmental factors distribution in the South China Sea (SCS). (**a**) Sampling stations and water masses. (**b and c**) Temperature and salinity of each station, respectively. Dashed arrow lines indicate the currents in the surface SCS during the summer (31). (**d**) Differences of environmental factors, including temperature (°C), salinity (PSU), nitrite + nitrate (NO_*x*_, µM), ammonia (NH_4_, µM), phosphate (PO_4_, µM), and silicate (SiO_3_, µM), among the water masses. All the values were ln-transformed. Different lowercase letters above each box in the same represent significant differences between groups (Tukey’s HSD test, *P* value < 0.05). Vietnam Warm Eddy Basin (VWEB); the Vietnam Cold Eddy Basin (VCEB); the Qiongdong Basin (QDB); the Pearl River Mouth Basin (PRMB); and the Luzon Warm Eddy Basin (LWEB). Map made with Natural Earth. Free vector and raster map data at naturalearthdata.com.

Our sampling covered the South China Sea (SCS), excluding the southeastern region (Fig. 1a). We collected a total of 68 water samples across five zones, each characterized by distinct physicochemical properties: 27 samples from NORC2018-06 (collected from 18 August 2018 to 21 September 2018), 20 samples from NORC2019-07 (collected from 23 June 2019 to 3 October 2019), and 21 samples from NORC2020-05 (collected from 19 July 2020 to 8 October 2020). Sampling depths ranged from 1.5 m to 10 m across an area spanning 110.27°E to 120.00°E and 9.33°N to 22.21°N. One liter of seawater was pre-filtered through a 20 µm nylon mesh (Sefar Nitex, Bigman AB, Sweden) and then filtered through a 0.22 µm pore size polycarbonate filter (Millipore, Billerica, MA, USA). The filters were immediately frozen in liquid nitrogen onboard and stored at −20°C until DNA extraction. For microbial abundance estimation, 2 mL samples were pre-filtered through a 20 µm nylon mesh, fixed with 1% (vol/vol) glutaraldehyde, incubated in the dark for 15 min, then frozen in liquid nitrogen, and stored at −20°C for later analysis.

### 16S rRNA gene sequencing and bioinformatic analysis

The phenol-chloroform-isoamyl alcohol method was applied to extract microbial DNA, as described previously (32). For the taxonomic identification of microbial compositions, the prokaryotic 16S rRNA V4-V5 fragments were amplified with forward primers (515F 5′-GTGCCAGCMGCCGCGGTAA-3′) and reverse primers (907R 5′-CCGTCAATTCMTTTRAGTTT-3′). Amplicons were quantified and sequenced using 2 × 250 bp paired-end reads on the Illumina MiSeq platform (Illumina, San Diego, CA, USA). Here, we used QIIME2-Deblur pipeline (33), and all sequences were trimmed to 225 bp with default parameters. The sequenced data set contained 2,651,200 sequences for 16S rRNA gene. The rarefaction of sample reads is performed by the qiime diversity core-metrics-phylogenetic command based on the minimum sample sequence number after denoise, the minimum sequence numbers for 16S rRNA were 18,789. The final data set contained 1,277,652 sequences comprising 3,730 amplicon sequence variants (ASVs) after removing the chloroplast or mitochondria for 16S rRNA gene.

### Environmental factor measurement

Water temperature and salinity were measured *in situ* with conductivity-temperature-depth oceanic profilers (SBE-911 Plus). Inorganic nutrients, including nitrite + nitrate (NO_*x*_), phosphate (PO_4_), and silicate (SiO_3_) concentrations, were assessed by the Technicon AA3 Auto-Analyzer (Bran-Luebbe, GmbH) (34). Moreover, ammonium concentrations (NH_4_) were measured by the orthophthaldialdehyde fluorometric method (35).

Based on satellite remote sensing data (Global Ocean Gridded L4 Sea Surface Heights and Derived Variables Reprocessed 1993 Ongoing), we conducted an analysis of the monthly mean sea level anomaly (SLA; defined as the difference between the observed sea surface height and the mean sea level) and the direction and velocity of geostrophic currents during the sampling period (July to September from 2018 to 2020, Fig. S1). These two parameters were combined to identify mesoscale eddies in the ocean.

### Microbial abundance measurement

For microbial abundance, thawed samples were stained with the nucleic acid-specific dye SYBR green I (Invitrogen, Carlsbad, CA, USA) for 15 min in a dark environment, after that they were analyzed using an Epics Altra II flow cytometer (Beckman Coulter, Inc., Brea, CA, USA) equipped with an external quantitative sample injector (Harvard Apparatus PHD 2000; Instech Laboratories, Inc.) following the protocols described previously (36).

### Co-occurrence network inference and analysis

Co-occurrence network was used to explore co-occurrence patterns of prokaryotes in the SCS. Only the ASVs occurred more than 1/4 of all sites were selected. A Spearman’s correlation between ASVs was considered statistically robust if the Spearman’s coefficient |*r*| > 0.6, and adjusted *P*-value was <0.05 (Benjamin-Hochberg method). A set of network properties was calculated to describe the topology of the resulting networks (37), including no. edges (*L*), no. positive edges (*L_p_*), no. negative edges (*L_n_*), no. nodes (*n*), connectance (Con), average degree (Ave. *K*), average path distance, diameter (*D*), average clustering coefficient (Ave. CC), centralization of degree (Centra. of degree), centralization of betweenness (Centra. of betweenness), and centralization of closeness (Centra. of closeness). A fast greedy modularity optimization algorithm was used to partition the modulesl the no. module and relative modularity (RM) were calculated based on modules. Then, the metric of within-module connectivity (*Z*_*i*_) and between-module connectivity (*P*_*i*_) were utilized to identify the connectors connecting distinct modules (37). Node can be categorized into four categories based on *Z*_*i*_ and *P*_*i*_ scores (37): (i) peripheral nodes (*Z*_*i*_ < 2.5, *P*_*i*_ < 0.62), (ii) connectors (*Z*_*i*_ < 2.5, *P*_*i*_ > 0.62), (iii) module hubs (*Z*_*i*_ > 2.5, *P*_*i*_ < 0.62), and (iv) network hubs (*Z*_*i*_ > 2.5, *P*_*i*_ > 0.62). All co-occurrence analyses were carried out using “igraph” (38) and “ggClusterNet” (39) packages. Network visualizations were conducted in Cytoscape version 3.8.0 and Gephi 0.9.2.

### Ecological mechanisms and niche breadth of prokaryotic community assembly

The phylogenetic bin-based null model analysis (iCAMP) was utilized with recommended default settings to calculate the nearest taxon index (NTI) and uncover the ecological drivers of prokaryotic community assembly (40) and to assess the relative influence of each ecological process on microbial community, including dispersal limitation (DL), homogenizing dispersal (HD), homogeneous selection (HoS), heterogeneous selection (HeS), and drift (DF). Moreover, the modified stochastic ratio (MST), which represents the relative contribution of stochasticity on prokaryotic community assembly, was calculated for each water mass using iCAMP.

Niche breadth is a key characteristic that shapes the balance between deterministic and stochastic processes in community assembly (41–43). Resource states were defined as the optimal categories of standardized seawater environmental variables across stations, determined through *K*-means partitioning using the cascadeKM function in the R package vegan (44). The number of resource states was identified based on the simple structure index (SSI) criterion, which integrates three elements: the maximum difference of each variable between clusters, the sizes of the most contrasting clusters, and the deviation of a variable’s cluster center from its overall mean. These elements collectively assess the interpretability of a partitioning solution, with the highest SSI value indicating the best partition (45). The number of resource states serves as a measure of habitat heterogeneity, where a larger number reflects more heterogeneous environmental conditions. Additionally, the niche breadth of prokaryotic amplicon sequence variants (ASVs), quantified using Levins’ niche breadth index (*B*), and community niche breadth (*B*_com_) were calculated based on the evenness of species abundance across different resource states, following methodologies outlined in previous studies (43, 46).

### Function prediction

Phylogenetic investigation of communities by reconstruction of unobserved states (PICRUSt2, version 2.3.0b) and functional annotation of prokaryotic taxa (FAPROTAX) were used to predict the functional potential of the bacterial community based on 16S rRNA gene sequencing profiles, following the developers’ guidelines (47, 48). The predictions from PICRUSt2 were mapped to the annotated gene catalog of the Kyoto Encyclopedia of Genes and Genomes (KEGG) database. FAPROTAX, on the other hand, translated the taxonomic profiles of the microbial community into putative functional profiles using a database of cultured microorganisms. The genes annotated by PICRUSt2 were subsequently enriched into relevant pathways using the clusterProfiler package in R (49).

### Statistics

Richness and Shannon indices of prokaryotic communities were calculated to evaluate the alpha diversity. A principal coordinate analysis (PCoA) based on Bray-Curtis dissimilarity was applied, and a permutational multivariate analysis (PERMANOVA) was conducted to assess the variance of prokaryotic community among all pairs of water masses. Variance portioning analysis (VPA) was conducted to explore the influence of environmental factors and geographic distance using vegan R package. Statistical analyses were performed in the R environment (v4.2.2; http://www.r-project.org/), and the packages “vegan,” “ggplot2,” and “ggpubr” were used without special comment (44, 50, 51).

The random forest model was applied to identify biomarkers for different water masses using the “randomForest” package in R (ntree = 1,000, with the default mtry of *p*/3, where *p* represents the number of ASVs) (52). Here, biomarkers are ASVs selected by the random-forest classifier because they best discriminate among water masses, whereas keystone taxa are nodes with high within-module (*Z_i_* > 2.5) or among-module (*P_i_* > 0.65) connectivity that uphold network integrity. Lists of ASVs ranked by feature importance were generated based on 100 iterations of the model. To determine the optimal number of marker taxa, we conducted 10-fold cross-validation with five repeats using the rfcv function from the same package. The minimum cross-validation error was achieved with 29 important ASVs (Fig. S2), and the error curve stabilized beyond this point. Therefore, we selected the 29 most important ASVs as marker taxa (biomarker), correlating with environmental factors.

## RESULTS

### Hydrological conditions of the South China Sea

The geostrophic currents in the SCS display intricate local variations, but the overall summer circulation is characterized by a dominant flow from the western coast of Vietnam toward the northeastern SCS (31), with increased dispersion as the current moves forward (Fig. 1). Additionally, satellite data showed the presence of a stable cold and warm eddy pair in the western SCS, with the warm eddy exerting a stronger influence near the Luzon Strait (Fig. S1). However, SLA heights in the northwestern SCS exhibited limited variability, showing no clear or consistent patterns of cold or warm eddies (Fig. S1). These results suggest the complicated hydrological conditions in the SCS.

Here, five water masses were identified based on temperature, salinity, and the locations (latitude and longitude) (temperature: 28.0–30.9°C and salinity: 32.9–33.9 PSU; Fig. 1 and Fig. S3). Furthermore, the water mass identification was corrected based on observed differences in SLA and geostrophic currents along the primary flow direction. These groups include the Vietnam Warm Eddy Basin (VWEB, 9 stations), the Vietnam Cold Eddy Basin (VCEB, 18 stations), the Qiongdong Basin (QDB, 13 stations), the Pearl River Mouth Basin (PRMB, 18 stations), and the Luzon Warm Eddy Basin (LWEB, 10 stations).

Among these water masses, the LWEB recorded the highest temperature (30.9 ± 0.53°C), while the VCEB had the lowest (28.0 ± 0.56°C) (Fig. 1). Salinity was lowest in the VWEB (32.9 ± 0.45 PSU), whereas the other four water masses exhibited higher, but similar, salinity values (~33.8 PSU). The inorganic nutrient concentrations varied across the five water masses. NO_*x*_, NH_4_, and PO_4_ were highest in the VCEB (1.13 ± 1.15 µM, 1.56 ± 1.16 µM, and 0.06 ± 0.02 µM, respectively), while the lowest NO_*x*_ was observed in the PRMB (0.17 ± 0.10 µM) and the lowest NH_4_ and PO_4_ in the QDB (0.30 ± 0.43 µM and 0.04 ± 0.03 µM, respectively). SiO_3_ ranged from 1.28 ± 0.53 µM in the VCEM to 3.16 ± 1.26 µM in the LWEB, generally increasing northward. In addition, environmental heterogeneity increased from south to north generally, suggesting that the environmental variation increased from south to north in the SCS. These findings suggest distinct hydrological conditions in each water mass, likely influencing the composition of prokaryotic communities within.

### Diversity of prokaryotic communities and microbial abundance

**Fig 2:**
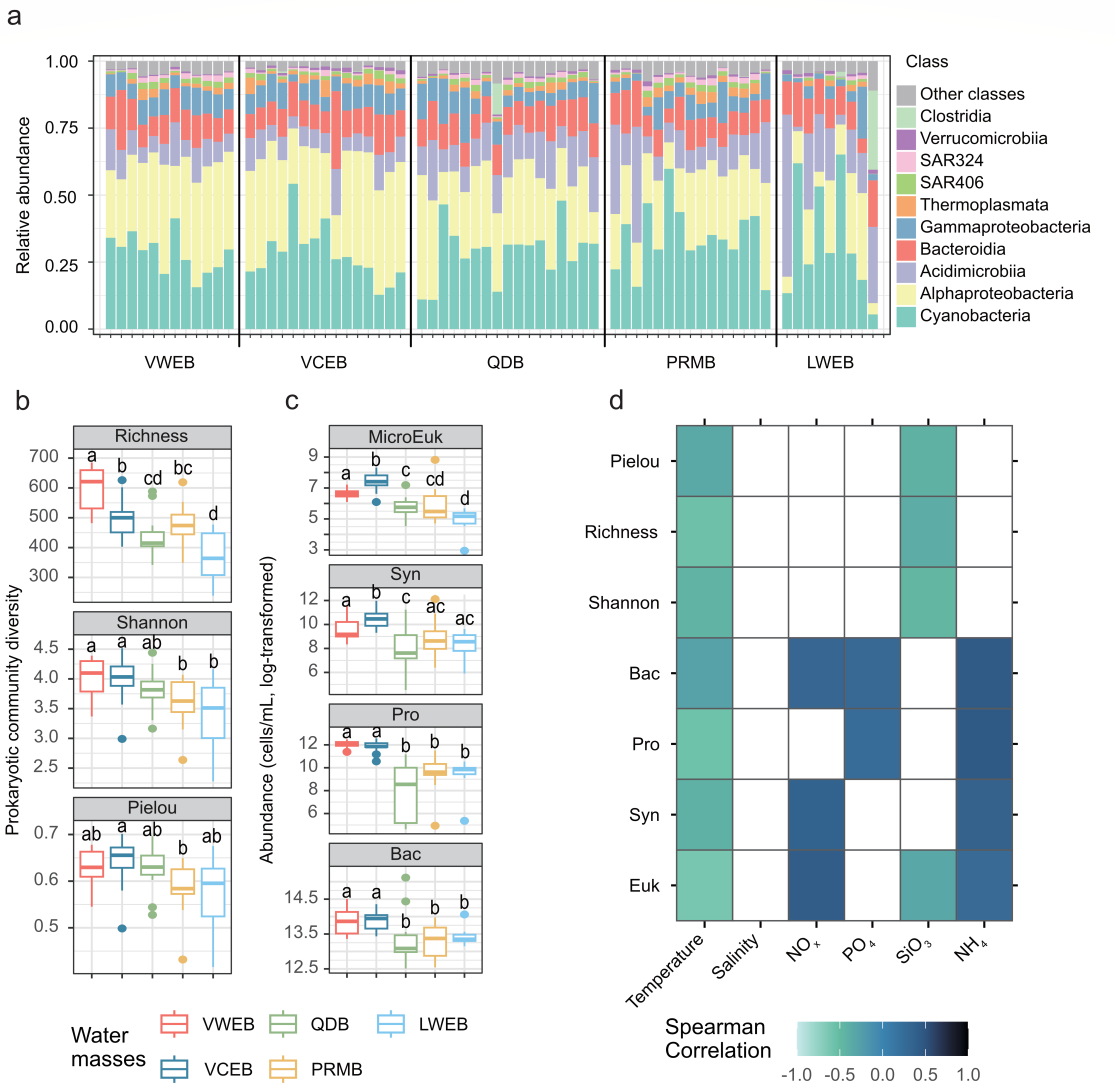
Taxonomic composition, abundance and alpha diversity of surface microbial communities in the South China Sea. (**a**) Taxonomic composition of prokaryotic communities at class level. The top 10 abundant classes were shown. (**b**) Alpha diversity of prokaryotic communities in the water masses, including richness, Shannon diversity, and Pielou’s evenness. Different lowercase letters above each box in the same represent significant differences between groups (Tukey’s HSD test, *P* value < 0.05). (**c**) Abundance of prokaryotes (cells/mL, ln-transformed) in the water masses, including microeukaryote (Euk), Synechococcus (Syn), Prochlorococcus (Pro), and bacteria (Bac). (**d**) Spearman correlations of abundance and diversity of microbes with environmental factors. Number and color indicate the Spearman correlation rho, while the white cell indicates the *P* value > 0.05.

The prokaryotic communities in the SCS are primarily composed of Cyanobacteria (accounted for total data set, 30.29%), Alphaproteobacteria (27.87%), Acidimicrobiia (13.10%), Bacteroidia (11.26%), and Gammaproteobacteria (7.32%) (Fig. 2a), collectively representing 89.84% of all prokaryotic communities across samples. Additionally, we observed an increasing trend in the proportions of Cyanobacteria and Acidimicrobiia along the ocean current from south to north (Table S1). Richness, Shannon diversity, and Pielou’s evenness also gradually declined along this south-to-north gradient (Fig. 2b), with significant correlations to temperature and SiO_3_ levels (Spearman correlation, *P* < 0.05) (Fig. 2b). These findings suggest a consistent pattern of alpha diversity changes in the prokaryotic communities along the SCS currents.

The abundance of microbes varied among the water masses (Fig. 2c; Table S2). Microeukaryotes peaked in the VCEM (1.88 ± 0.97 × 10³ cells mL⁻¹), declined in the VWEB (0.81 ± 0.29 × 10³ cells mL⁻¹) and PRMB (0.82 ± 1.76 × 10³ cells mL⁻¹), and reached their minimum in the LWEB (0.17 ± 0.10 × 10³ cells mL⁻¹). *Synechococcus* and *Prochlorococcus* displayed a similar pattern: both were most abundant in the VCEB (4.85 ± 4.08 × 10⁴ and 1.54 ± 0.61 × 10⁵ cells mL⁻¹, respectively) and the VWEB (2.45 ± 3.02 × 10⁴ and 1.71 ± 0.46 × 10⁵ cells mL⁻¹), but least abundant in the QDB (0.96 ± 1.89 × 10⁴ and 0.17 ± 0.24 × 10⁵ cells mL⁻¹) and LWEB (0.64 ± 0.54 × 10⁴ and 0.18 ± 0.12 × 10⁵ cells mL⁻¹). Total bacterial counts were highest in the VWEB (1.11 ± 0.40 × 10⁶ cells mL⁻¹) and lowest in the PRMB (0.64 ± 0.29 × 10⁶ cells mL⁻¹).

Significant correlations of diversity and microbial abundances with environmental factors were observed in our study (Fig. 2d). However, only temperature and SiO_3_ showed significant correlations with the alpha diversity of prokaryotic communities. Instead, we found a strong negative correlation between microbial abundances and temperature (Spearman correlation rho = −0.61, −0.41, −0.56, and −0.28 for microeukaryotes, *Synechococcus*, *Prochlorococcus,* and bacteria, respectively, *P* values < 0.05). Nevertheless, no significant correlation was found between microbial abundances and salinity (Spearman correlation, *P* values > 0.05), suggesting a limited influence of salinity. For the inorganic nutrients, NO_*x*_ showed significantly positive correlations with the abundances of microeukaryotes (rho = 0.43, *P* value < 0.01), *Synechococcus* (rho = 0.37, *P* value = 0.01), and bacteria (rho = 0.33, *P* value = 0.02). PO_4_ was positive correlations with *Prochlorococcus* (rho = 0.26, *P* value = 0.04) and bacteria (rho = 0.30, *P* value = 0.01). NH_4_ was positively correlated with abundances of all four microbial groups (microeukaryotes: rho = 0.29, *P* value = 0.02; *Synechococcus:* rho = 0.37, *P* value < 0.01; *Prochlorococcus:* rho = 0.45, *P* value < 0.01; bacteria: rho = 0.45, *P* value < 0.01). In contrast, SiO_3_ was significantly correlated only with microeukaryotic abundances (rho = −0.32, *P* value = 0.01). Overall, these results suggest that temperature and inorganic nutrient levels within the water masses play a significant role in influencing prokaryotic community diversity and microbial abundances in the SCS.

### Distance decay of prokaryotic communities on geographic and environmental distances

**Fig 3:**
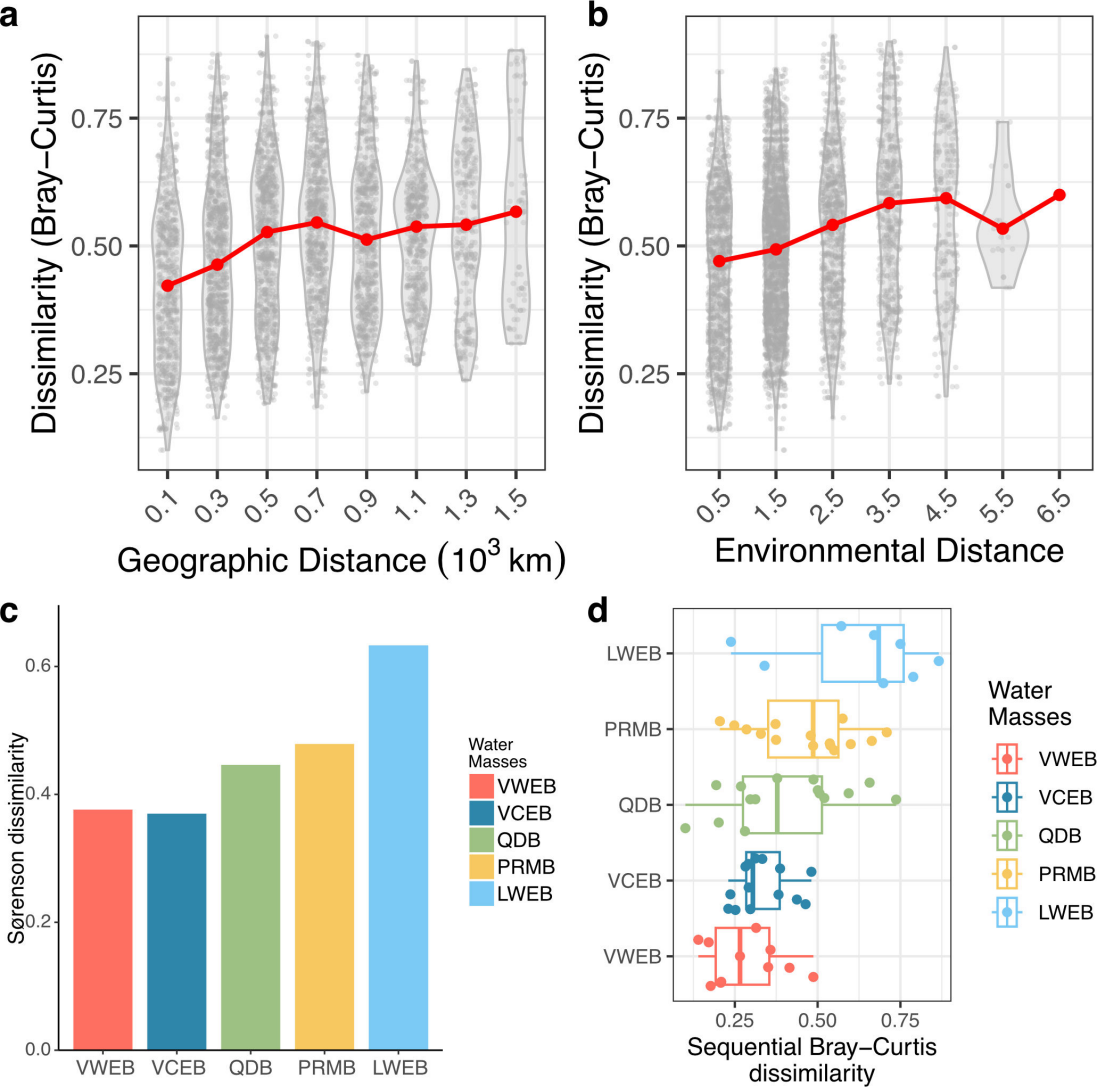
Beta diversity and environmental drivers of surface prokaryotic community composition. (**a and b**) Distance decay of surface SCS prokaryotic communities based on geographic distance (**a**) and environmental distance (**b**). Pairwise prokaryotic community dissimilarity (Bray-Curtis) were calculated based on relative ASV abundances increases with distance (geographic and environmental distances) between sampling stations. Environmental distances were calculated based on temperature, salinity, NO_*x*_, NH_4_, PO_4_, and SiO_3_. (**c**) Sørenson dissimilarity of surface prokaryotic communities within each water mass. (**d**) Sequential Bray-Curtis dissimilarities for surface prokaryotic communities, along with means were significantly different between domains (Wilcoxon test, *P* values < 0.05).

**Fig 4:**
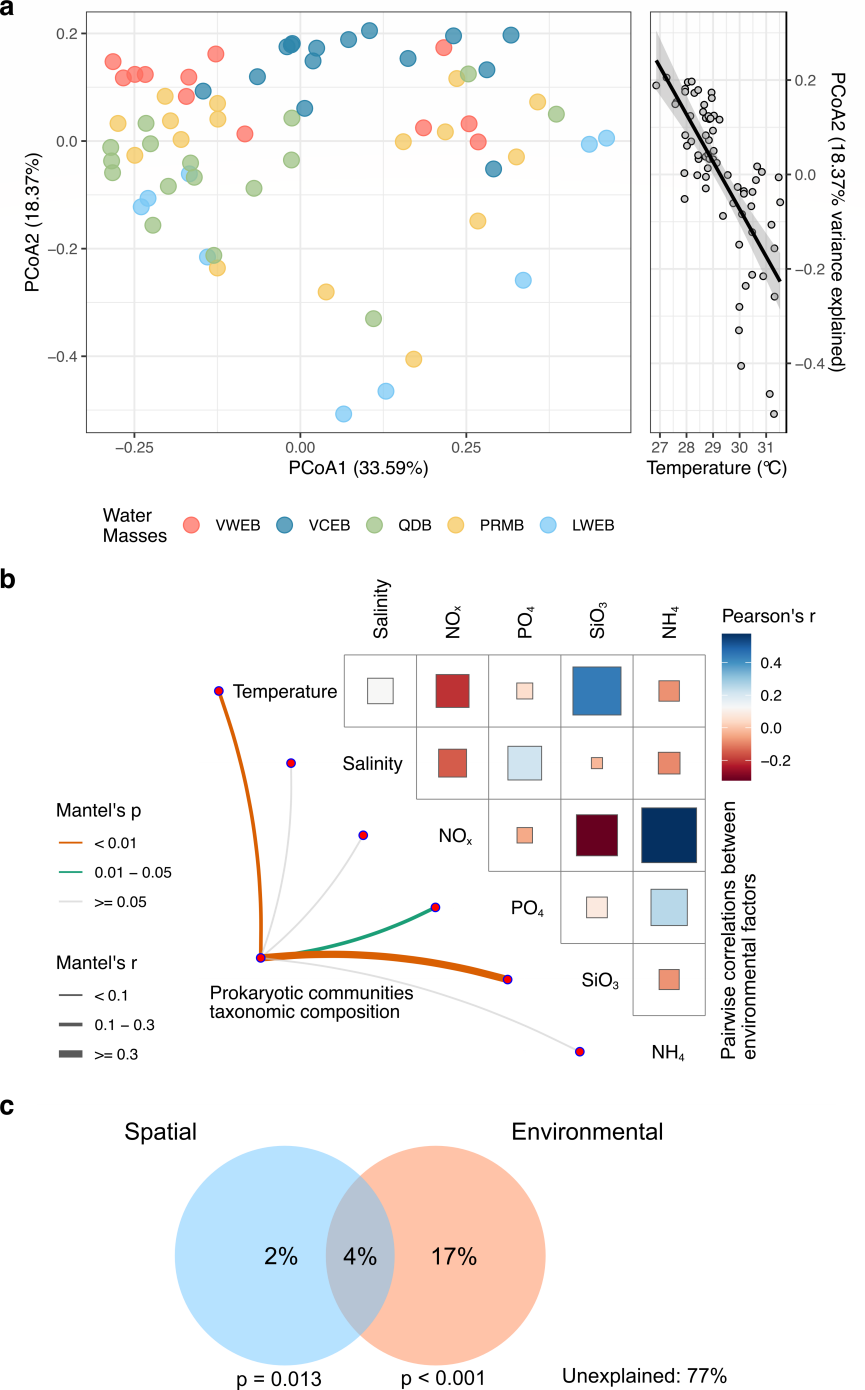
Environmental drivers of surface prokaryotic community composition. (**a**) Principal coordinate analysis (PCoA) of surface samples shows that samples are not clearly grouped by their regional origin (left), but rather separated by the local temperatures as shown by the strong correlation (adjusted *R*^2^: 0.51) between the second PCoA and temperature (right). (**b**) Pairwise comparisons of environmental factors are shown, with a color gradient denoting Pearson’s correlation coefficient. Taxonomic community composition was related to each environmental factor by partial (geographic distance–corrected) Mantel tests. Edge width corresponds to Mantel’s *r* statistic for the corresponding distance correlations, and edge color denotes the statistical significance based on 9,999 permutations. (**c**) Variance portioning analysis on Bray-Curtis dissimilarity of prokaryotic communities showing the relative contribution of spatial and environmental factors alone and in combination. Partial *R*^2^ values are provided for each fraction. Blue cycle represents spatial structure, and orange circle represents environmental factors. The *P* values are obtained using redundancy analysis for spatial and environmental factors, respectively, with 999 permutations.

We found that prokaryotic community dissimilarity increased with both geographic and environmental distance in the SCS (Fig. 3a and b), indicating that community differences across samples were largely due to spatial species turnover. Additionally, we found the Sørenson and sequential dissimilarity within individual water masses rose from south to north (Fig. 3c and d), suggesting the increased spatial variation along the current. A PCoA of taxonomic compositions did not show clear separation by water mass (Fig. 4a), though significant structural differences were observed across water masses (Table S3). Instead, temperature was strongly correlated with PCoA2 (Pearson’s correlation, adjusted *R*² =0.51, *P* < 0.01). To assess the geographic independence of these patterns and identify environmental drivers, we examined correlations between distance-corrected taxonomic dissimilarities and environmental factors (Fig. 4b). Temperature emerged as the strongest correlation of taxonomic composition (Mantel *R* = 0.21, *P* < 0.01), while salinity showed no significant correlation (Fig. 4b). Most nutrients had weak correlations with prokaryotic communities, except for SiO_3_. Despite the influences of environmental factors varied across the water masses, temperature appears to be a significant driver in most water masses, with other factors such as salinity and nutrients (PO_4_ and SiO_3_) playing variable roles across the basins (Fig. S4). Furthermore, VPA revealed that environmental factors explained 17% of the variation in prokaryotic communities, with spatial location accounting for only 2% (Fig. 4c). However, a substantial portion of variation (77%) remained unexplained by either environmental factors or spatial location.

### Difference in niche breadth of prokaryotic communities between water masses

Based on SSI derived from *K*-means partitioning, the environmental conditions across stations were classified four, three, four, six, and three resource states of each water mass, respectively (Fig. S5). In general, the Levins’ niche breadth of prokaryotic communities (*B*_com_) was higher in the VWEB, VCEB, and QDB compared to PRMB and LWEB (Wilcoxon test, adjusted *P* values < 0.001). Additionally, *B*_com_ of the PRMB was significantly higher than that in the LWEB (adjusted *P* value < 0.001), whereas no significant differences were detected among the VWEB, VCEB and QDB (adjusted *P* values > 0.05; Fig. S6).

### Assembly process of prokaryotic communities

**Fig 5:**
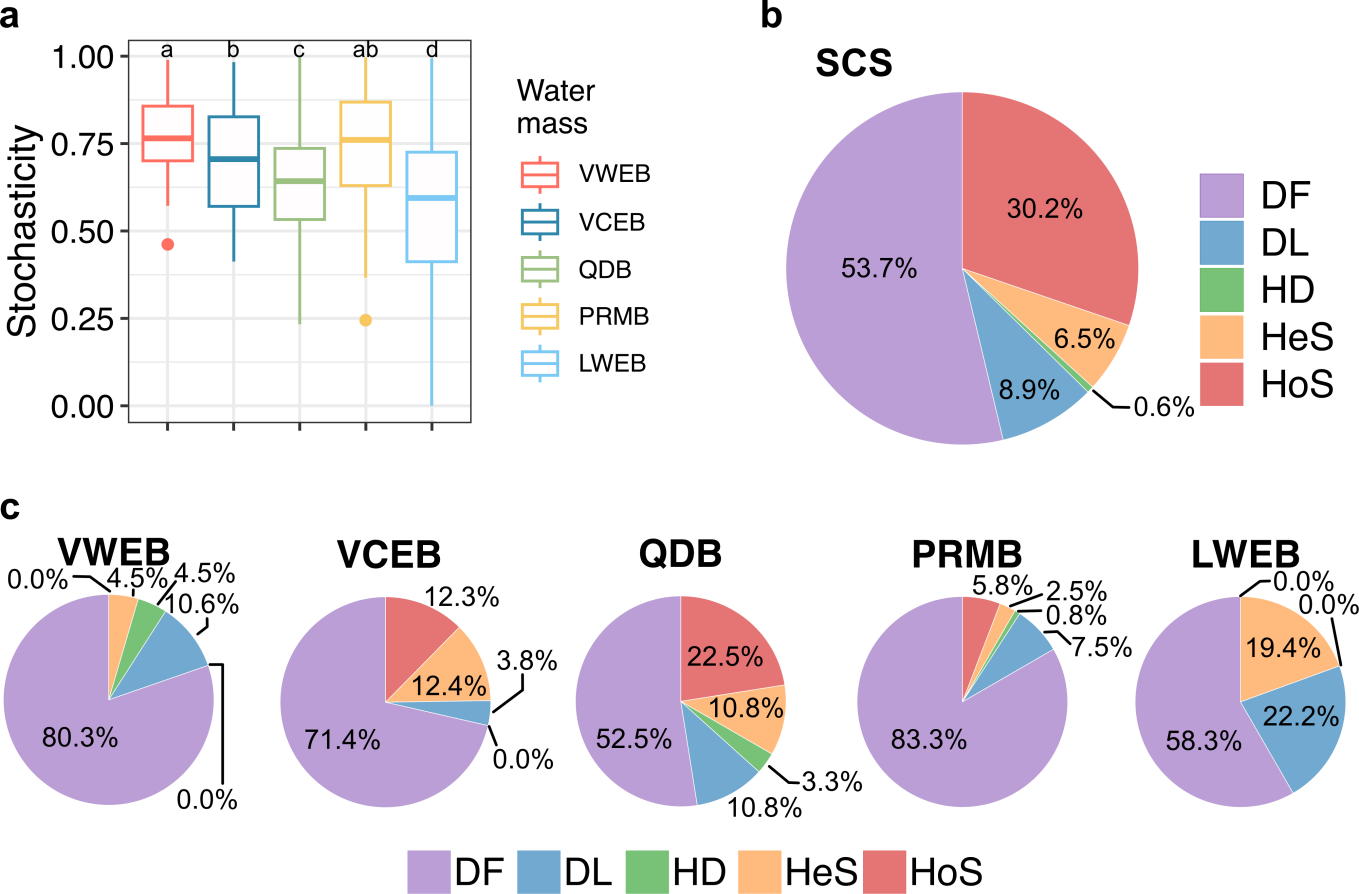
Prokaryotic community assembly processes. (**a**) Modified stochasticity ratio of prokaryotic communities in the SCS. Different lowercase letters above each box in the same represent significant differences between water masses (Tukey’s HSD test, *P* value < 0.05). (**b**) Relative proportion of ecological processes for the prokaryotic community across all stations in the SCS. Five different ecological processes (homogeneous selection [HoS], heterogeneous selection [HeS], drift [DF], dispersal limitation [DL], and homogeneous dispersal [HD]) were analyzed. (**c**) Ecological processes for the prokaryotic community among different water masses in the SCS.

MST of prokaryotic communities decreased from south to north in the SCS (Fig. 5a). Overall, ecological drift emerged as the dominant mechanism of driving community assembly, accounting for 53.7% (Fig. 5b). Environmental selection, encompassing HeS and HoS, contributed 38.7% of relative importance, while dispersal, including DL and HD, accounted for only 9.5%. Within individual water masses, drift remained the primary driver of community assembly but showed a gradual decline in influence from south to north, except in the QDB (Fig. 5c). However, other processes showed no clear north-south trend. Environmental selection was notably higher in the QDB than in other water masses, while DL reached its highest value in the LWEB.

### Co-occurrence network of prokaryotic communities

**Fig 6:**
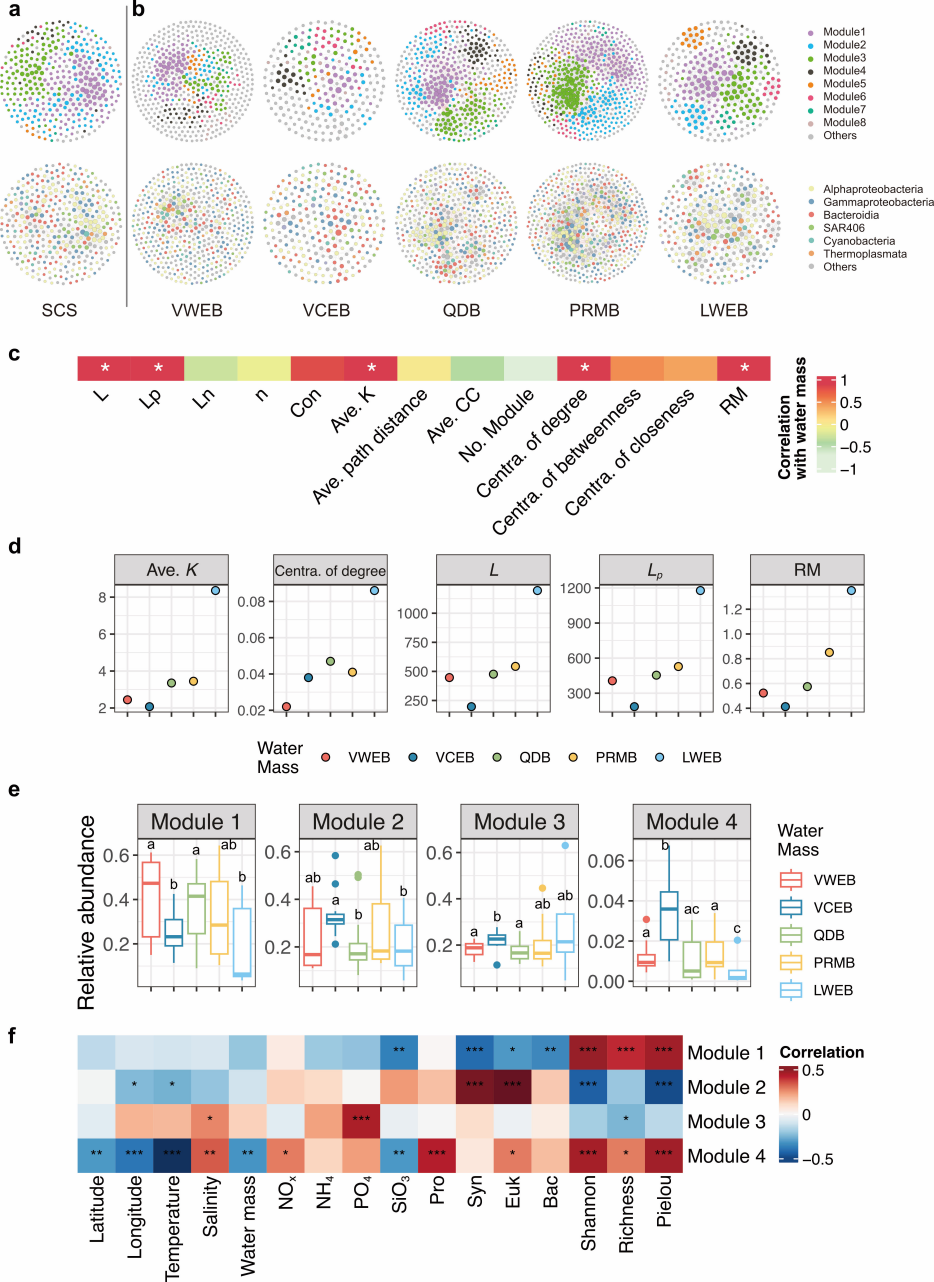
Succession of prokaryotic networks over water masses. (**a**) Co-occurrence networks of prokaryotic communities in the South China Sea. (**b**) Subnetworks in each water mass. A connection indicates a strong (Spearman’s |*r|* ≥ 0.6) and significant (FDR-corrected *P*-value < 0.05) correlation. Colors in the upper six networks indicate the module, while the ones in the lower six networks indicate the class of ASVs. (**c**) Spearman correlation between subnetwork properties and water masses (from south to north). The asterisk indicates the *P* value < 0.05. (**d**) Changes of network topology, including Ave. *K*, Centra. of degree (centralization of degree), *L*, *L_p_* and RM, which were significantly correlated with water masses showing in panel c. (**e**) Differences in relative abundances of dominant co-occurrence modules among different prokaryotic communities. Different lowercase letters above each box in the same panel represent significant differences between water masses (Tukey’s HSD test, *P* value < 0.05). (**f**) Heatmap showing Spearman correlations between co-occurrence modules and environmental factors. *, *P* value < 0.05; **, *P* value < 0.01; ***, *P* value < 0.001.

To highlight the role of biotic associations in shaping community variation, we constructed a co-occurrence network for the SCS region (Fig. 6a) and water masses along with five subnetworks, each specific to one water mass (Fig. 6b). This SCS network comprised 353 nodes and 1,712 edges, with 90.48% of edges indicating positive associations and 9.52% negative (Table S4). Notably, over one-third of these edges involved Alphaproteobacteria (638 edges), followed by Gammaproteobacteria (319 edges), Bacteroidia (181 edges), and SAR406 (132 edges). Among individual ASVs, *asv3925* (Cyanobacteria) displayed the highest connectivity, likely due to its autotrophic nature, while Thermoplasmata also exhibited substantial connectivity, suggesting unique ecological roles among archaea, ranking just below Alphaproteobacteria and Gammaproteobacteria.

Several topological properties of the subnetworks, including *L*, *L_p_*, Ave.*K*, degree of centrality, and RM, showed significantly positive correlations with both water mass and the latitudinal gradient from south to north (Fig. 6c and d). Additionally, four major modules (modules 1–4) were identified based on the node number (>10). The relative abundance of ASVs within modules 1, 2, and 4 generally declined from south to north (Fig. 6e), whereas ASVs in module 3 increased in abundance along this gradient. Modules 1, 2, and 4 were significantly associated with prokaryotic community diversity (Fig. 6f); however, modules 1, 2, and 3 displayed weaker correlations with environmental factors compared to module 4, suggesting lower sensitivity of these modules to environmental changes. Overall, these results highlight modular shifts in prokaryotic community composition across different water masses in the SCS.

### Keystone species and prokaryotic biomarkers

**Fig 7:**
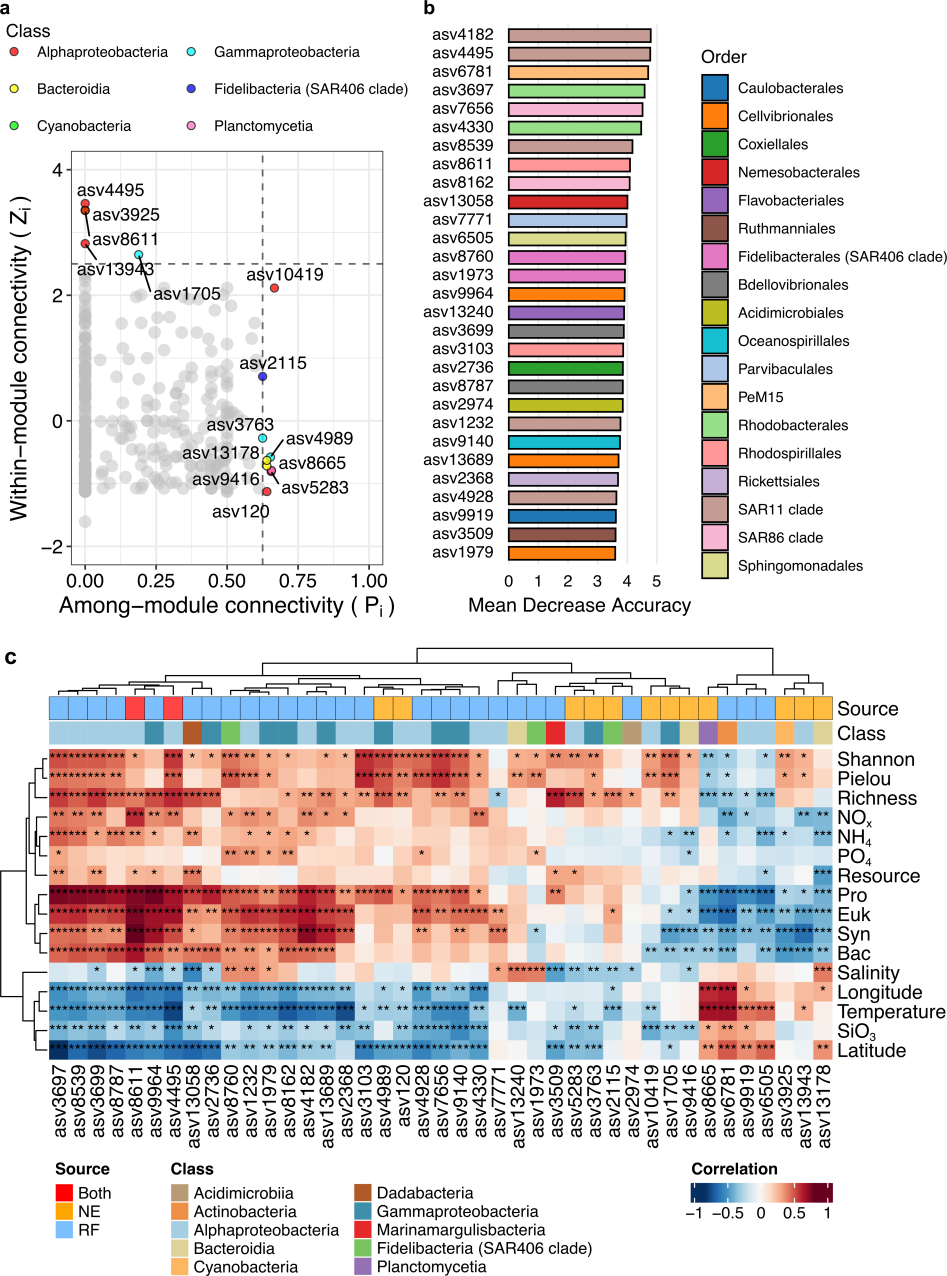
Keystone species and biomarkers. (**a**) Keystone species was identified by within-module connectivity (*Z*_*i*_) and among-module connectivity (*P*_*i*_). Gray dots indicate hyper. The dots with *Z*_*i*_ > 2.5 and *P*_*i*_ < 0.65 are the module hubs, while the dots with *Z*_*i*_ < 2.5 and *P*_*i*_ > 0.65 are the connectors. (**b**) Top 29 ASVs were identified using random forest model. The higher means decrease accuracy indicates the more importance of ASV. The random forest model was tested by 100 iterations and revealed a *P* value < 0.05. (**c**) Spearman correlations between keystone species and environmental factors. Source indicates the ASV identified by network (NE), random forest model (RF), or both. The Class color indicates the class of these ASVs. *, *P* value < 0.05; **, *P* value < 0.01; ***, *P* value < 0.001.

We identified potential keystone species within the network by categorizing nodes into four groups—network hubs, module hubs, connectors, and peripherals—based on their *Z*_*i*_ and *P*_*i*_ values (Fig. 7a) (see Materials and Methods for details). Given their structural importance in network topology, module hubs and connectors were designated as keystone taxa. Based on these criteria, prominent keystone taxa included members of Alphaproteobacteria (six ASVs), Gammaproteobacteria (three ASVs), Bacteroidia (two ASVs), the SAR406 clade (one ASV), Cyanobacteria (one ASV), and Planctomycetia (one ASV). Their relative abundance ranged from 0.003% to 11.8% to total sequence number. Additionally, a random forest model was used to identify biomarkers specific to each water mass, yielding an out-of-bag (OOB) error rate of 22%, indicating a reliable model. Biomarker ASVs exhibited diverse taxonomy, including the SAR11 clade (five ASVs), Cellvibrionales (three ASVs), SAR86 clade (two ASVs), and SAR406 clade (two ASVs) (Fig. 7b). Their proportion ranged from 0.002% to 1.16% to total sequence number.

Two major clusters of these key species (both keystone taxa and biomarkers) were identified based on their correlations with environmental factors (Fig. 7c). For example, asv6781 (Actinobacteria), asv9919 (Alphaproteobacteria), and asv6505 (Alphaproteobacteria) showed positive correlations with temperature but negative correlations with nutrients such as NO_*x*_, NH_4_, and microbial abundance. In contrast, several ASVs, including asv4182, asv4495, and asv3697 (all Alphaproteobacteria), were negatively correlated with temperature but positively correlated with nutrients. Most biomarker ASVs were positively correlated with diversity, suggesting a contributory role in enhancing community diversity.

### Function of prokaryotic community

**Fig 8:**
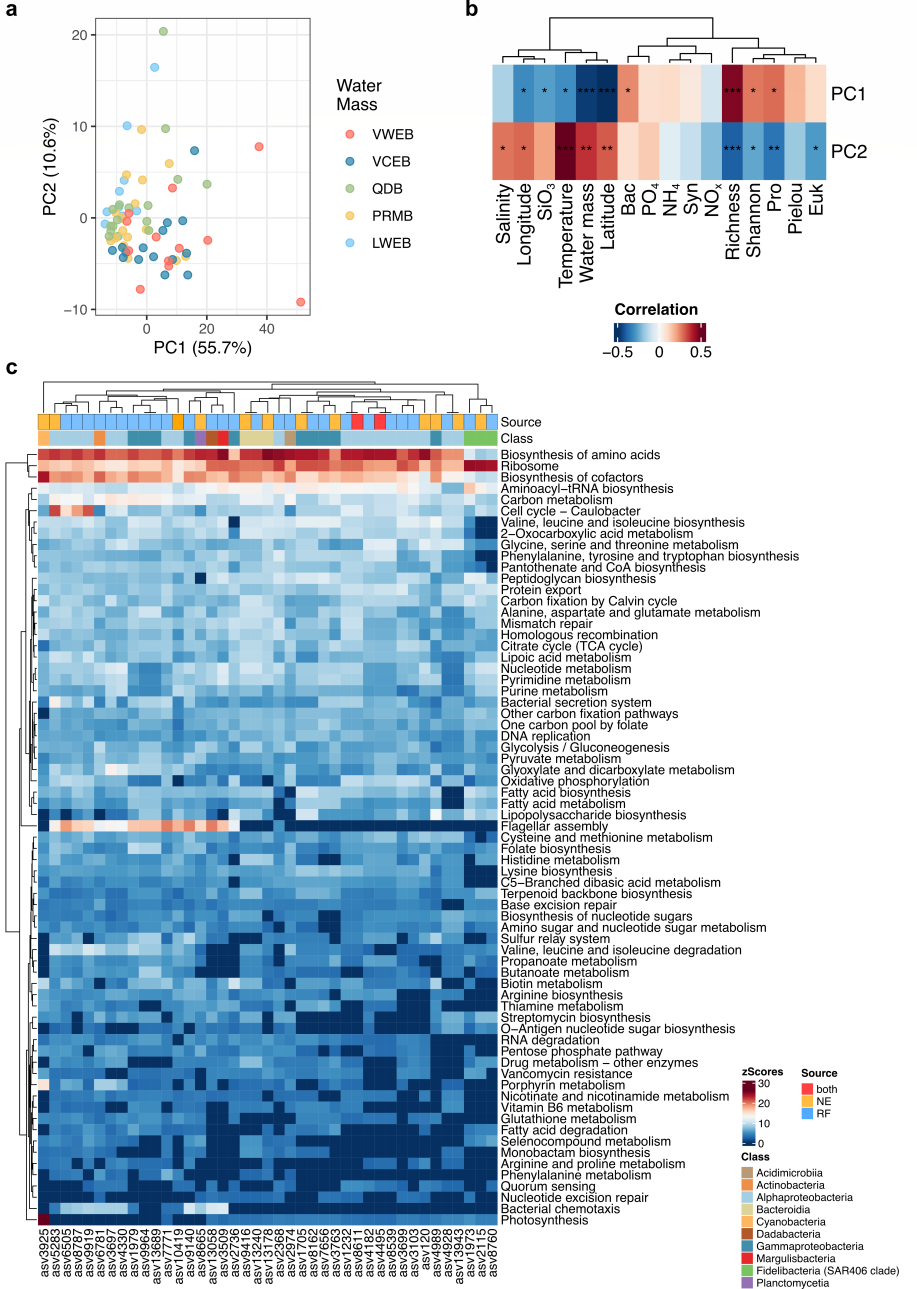
Functional profile of prokaryotic communities and key species. (**a**) Principal coordination analysis (PCA) of the function of prokaryotic communities based on PICRUST2 prediction. (**b**) Spearman correlations of PC1 and PC2 with environmental factors and diversity of prokaryotic communities. *P* values were adjusted using the FDR method. *, adjusted *P* value < 0.05; **, adjusted *P* value < 0.01; ***, adjusted *P* value < 0.001. (**c**) Metabolism enrichment of biomarkers and keystone species (adjusted *P* < 0.05). Only metabolism with more than half ASVs was shown here. The function was predicted based on PICRUSt2. Abundance of each metabolism was transformed by *z*-score. NE represents the ASV identified by network, while RF represents the ASV identified by random forest model. Both represent the ASV were identified by both NE and RF. Class shows the class of these ASVs belonging.

The PCA analysis revealed functional differences in prokaryotic communities, predicted using PICRUSt2, across the water masses (Fig. 8a). Notably, PC1 displayed positive correlations with Bac and Pro, as well as the richness and Shannon index of prokaryotic communities, while showing negative correlations with geographical location (longitude and latitude), SiO₃, temperature, and water mass classifications (Fig. 8b). In contrast, PC2 exhibited positive correlations with salinity, location, temperature, and water mass, but negative correlations with Euk and Pro, as well as the richness and Shannon index of prokaryotic communities. These findings suggest that variations in the functional composition of prokaryotic communities are closely associated with environmental changes in the SCS water masses.

Furthermore, the metabolism enrichment of the keystone species and biomarkers revealed that general functions of these species were similar, such as biosynthesis of amino acids, ribosome, biosynthesis of cofactors carbon metabolism (Fig. 8c; Fig. S8). However, distinct patterns of functional contributions were observed among different microbial taxa, with certain functions being more enriched in specific sources or classes. For instance, flagellar assembly and chemotaxis were predominantly enriched in the biomarkers, while photosynthesis was more prominently associated with the keystone species. These findings highlight the functional divergence across their ecological roles and suggest a potential link between prokaryotic community composition and environmental conditions.

## DISCUSSION

Hydrodynamic conditions, such as ocean currents and swell waves, are well-recognized drivers of marine microbial dispersal and migration, affecting both local and large-scale distribution patterns (11, 53). These physical forces not only facilitate the movement of microbial populations but also create spatial heterogeneity by altering nutrient availability and environmental conditions. The impact of current-induced dispersal on prokaryotic communities has been studied in both freshwater and marine microbial ecosystems (53, 54). Here, we provide a systematic comparison of prokaryotic communities in the semi-enclosed SCS basin, emphasizing the significant role of water mass and ocean currents play in shaping community structure.

We observed a clear gradient in shifts of both alpha and beta diversity along the trajectory of the SCS current (Fig. 2 and 3), suggesting that ocean currents influence microbial dispersal. There are two hypotheses to describe this effect: the “dispersal mechanism” and the “bulk transport hypothesis” (55). The dispersal mechanism posits that advection mainly affects the presence and diversity of taxa, with minimal impact on abundance, whereas the bulk transport hypothesis suggests that advection primarily alters taxa abundance (55). Our findings revealed that geographic distance had a more pronounced effect on the presence-absence dissimilarity of prokaryotic communities (Sørensen dissimilarity, 0.49 ± 0.08) compared to abundance-based dissimilarity (Bray-Curtis dissimilarity, 0.32 ± 0.06) (Table S5). This pattern supports the dispersal hypothesis, suggesting that advection primarily influences microbial community structure by increasing colonization opportunities (55). Additionally, we found the high and decreased contribution of ASVs in the VWEB to other water masses (Fig. S9). Our findings, therefore, suggest that microbes can travel long distances via ocean currents, significantly shaping community structures (10, 56, 57). These patterns underscore the strong role of ocean currents in structuring microbial populations and shaping biodiversity gradients in the SCS (58, 59).

A meta-analysis of studies on microbial community composition showed that distance effects accounted for 10% of the variance, while environmental factors explained 27%, suggesting a stronger influence of contemporary environmental conditions over historical processes like dispersal limitation (60). Our findings align with this pattern, with dispersion processes, including dispersal limitation and homogenizing dispersal, explaining around 10% of the variance in the SCS. However, the variance attributed to geographic distance (2%) and environmental factors (17%) in our study was lower than previous estimates (Fig. 4c), possibly due to the smaller spatial scale. Similar trends have been observed in the Southern Ocean, where geographic distance had a minimal effect (55). Although incorporating the main physiochemical and spatial variables, our VPA left 77% of the community variance unexplained. This residual likely reflects ecological forces that were not quantified in the present survey, such as the molecular complexity of DOM, virus-mediated infection and lysis cycles, and centimeter-scale physical heterogeneity that creates patchy viscous seascapes (61). Despite the conservative estimate of the advection effect, it remains significant, indicating that physical transport plays an important role in microbial biogeography (10, 56, 57). Furthermore, a previous study has been reported the dynamic currents and diverse water mass composition in the SCS (62), highlighting the importance of understanding how physical processes shape microbial distribution at both global and regional scales.

Regionally, microbial communities can be influenced by local water masses along the current in at least two ways (63). First, water masses serve as a barrier to dispersal, influenced by differences in water density (64). Second, they act as a selective force due to variations in temperature, salinity (65), and organic matter composition (66–68). The negligible effect of salinity in our models is consistent with the very small range observed (32.9–33.9 PSU; SD = 0.3 PSU), which provides insufficient osmotic or ionic contrast to drive marked community differentiation. However, we found significant correlations between prokaryotic community structure and temperature in the SCS (Fig. 4), which aligns with previous studies that found bacterial communities associated with specific water masses (64, 66). Furthermore, a stronger Mantel correlation between environmental distance and prokaryotic communities, compared to geographic distance, supports this observation (Table S5). These findings lead us to further analyze how selection pressures within water mass shape the prokaryotic communities.

Selection pressures, encompassing both heterogeneous and homogeneous processes, varied across different water masses in our study (Fig. 6). The temperature is the most important driver of diversity globally (53). Likely, we found the significant influence of the temperature on the structure and diversity, as well as the function, of the prokaryotic communities. In contrast, the declined influence of nutrients on structure compared to temperature might relate to low concentrations of oligotrophic area studied. Furthermore, the different responses of prokaryotic communities are likely linked to different types of organic matter in each water mass (69–72), which likely select for different prokaryotes (66, 67). The significant correlation of prokaryotic community with SiO_3_ partially support this, which is one of the factors controlling phytoplankton distribution, especially the diatom in the SCS (73). Overall, these results suggest that local and regional environmental factors exert differential influences on microbial communities distributed along the current (74, 75).

Ecological drift also plays a predominant role in shaping the assembly of prokaryotic communities in the surface SCS, even though selection contributes significantly. Such findings align with studies conducted in the North Pacific Ocean, which also revealed high contribution of drift on microbial community assembly (76). This drift, arising from stochastic variations in population sizes, birth rates, and death rates (77), can have diverse impacts on bacterial dynamics (78). Previous studies have assumed that communities with relatively large population sizes would be less influenced by random births and deaths and thus less impacted by ecological drift, which may then influence the strength of dispersal limitation that operates alongside drift (43, 46). Supporting this, we found decreased abundances of heterotrophic bacteria, Prochlorococcus, and Synechococcus along the current from south to north, companying decreased drift effect. Moreover, the increase of habitat heterogeneity across the water masses from south to north (Fig. S5 and S6), could lead to higher habitat preference and, thus, less importance of stochasticity over determinism (79). Therefore, the spatial changes in prokaryotic niche breadths and habitat heterogeneity along the current could be related to the local variations of determinism-stochasticity in assembly mechanisms. In turn, the high contribution of ecological drift implies the well mixed water mass in the surface SCS, similar to patterns in river ecosystems (80, 81). Given seasonal changes in current directions (31), future studies should focus on assessing the impact of these directional changes on the assembly processes of marine microbiota.

Unlike macroorganisms, where individual populations simply accumulate, microorganisms in natural ecosystems form complex ecological networks critical for maintaining ecosystem functions (82). Biotic interactions play a crucial role in species sorting, thereby influencing microbial community assembly and biogeographic patterns (83). The co-occurrence network revealed an increased complexity of interactions among prokaryotes, as evidenced by the rise in the *L*, *L_p_*, Ave. *K*, Contra. of degree and RM values from south to north (Fig. 5), which also indicates the increase in stability (84). Microbial interactions could also be reshaped by gaining adaptive genes to extend niche breadth, which alters interaction patterns (85). The distinct functional structure of the prokaryotic communities might partially support this phenomenon (Table S6). While the prokaryotic communities along the current display highly redundant functional potential, evidenced by insignificant differences in functional diversity (ANOVA test, *P* = 0.51; Fig. S10), we observed that the network’s keystone species exhibit distinct metabolic functions. These keystone species are adapting to a variety of environmental conditions by expanding their metabolic capabilities (Fig. 8; Fig. S8), leading to altered interaction patterns and increased network complexity. Meanwhile, since we found significantly negative correlations between MST and several network properties (e.g., *n*, Con, Ave. *K*, and RM) (Table S7), selection likely amplifies the complexity of interactions among bacterial communities (Fig. 6), thereby contributing to greater overall stability (84).

Previous studies have shown the existence of environmentally driven modules, such as water depth (86) or soil properties (87). We found that the modules dominated by abundant ASVs (modules 1 and 2) were less affected by the environment, whereas the rare species modules (modules 3 and 4) were significantly affected (Fig. 7). Rare taxa, known for enhancing ecosystem stability across diverse environments (88–90), played key roles in nitrogen cycling (e.g., nitrate reduction and ammonification pathways predicted by PICRUSt2; Fig. 8), akin to patterns observed in soils (91). In contrast, modules 1 and 2 correlated with photoautotrophic metabolisms (Fig. S7), suggesting their role in carbon cycling. Given that modules in microbial co-occurrence networks may represent different niches (92), the present patterns of modules may also indicate a change in niches among the water masses. These module-specific functions indicate niche differentiation driven by ocean currents, influencing microbial interactions, functionality, and community stability in the SCS.

The response of specific species to different water masses varied based on their ecological traits (58). Keystone species made greater contributions to ecosystem functions (93), with 14 identified through network analysis and 29 biomarker ASVs identified via random forest analysis (Fig. 7). Stochastic processes, particularly ecological drift, had significant impacts on these species (Fig. S11), may relate to their metabolisms (e.g., fatty acid degradation and nucleotide metabolism) (Fig. S12). While both keystone species and biomarkers were affected by stochasticity, biomarkers were more strongly shaped (82% influence) than keystone species (60%). The limited overlap between biomarkers and keystone species suggests that distinct processes shape their composition and interactions, emphasizing the important role of stochastic factors in microbial community dynamics under environmental variability (94). The difference in the flagellar assembly ability of biomarkers with keystone species indicates that the mobility of prokaryotes influences their ability to respond rapidly to environmental changes, making them more prevalent as biomarkers. In contrast, keystone species may perform their critical ecological roles independently of mobility traits. This underscores that motility can enhance a microorganism’s responsiveness to environmental variability, thereby influencing its role as a biomarker, while keystone species contribute to community stability through other mechanisms. Additionally, most ASVs exhibiting motility possess bacterial chemotaxis abilities, whereas those without motility are capable of photosynthesis. The study of phytoplankton in the western Pacific Ocean shows that the mobility of Trichodesmium helps it adapt to environmental changes, similar to how motility aids biomarkers, while their photosynthetic ability parallels the role of non-motile ASVs (95). Therefore, the differential impacts of stochastic processes on biomarkers and keystone species, attributable to their distinct ecological traits, particularly motility and photosynthetic capabilities, highlight the crucial role these traits play in shaping microbial community dynamics and determining species-specific responses under environmental variability.

### Conclusions

This study demonstrates that ocean currents and environmental factors, especially temperature, are key drivers shaping prokaryotic community structure and function in the SCS by facilitating microbial dispersal and influencing diversity patterns along the current. While ocean currents enhance colonization opportunities supporting the dispersal mechanism hypothesis, temperature acts as a critical selection pressure affecting community composition. Stochastic processes like ecological drift also play a significant role, indicating a dynamic interplay between deterministic and stochastic factors in community assembly. Increased complexity and stability of microbial interactions are observed along the current, with niche differentiation driven by environmental selection and rare taxa contributing to ecosystem stability and nitrogen cycling. The differing responses of keystone species and biomarkers to environmental variability, due to traits like motility and photosynthetic capabilities, affect their ecological roles. Understanding these combined effects is essential for predicting microbial community dynamics under environmental variability, with important implications for ecosystem management and conservation.

## Data Availability

Altimeter satellite gridded sea level anomalies were collected from Global Ocean Gridded L4 Sea Surface Heights and Derived Variables Reprocessed 1993 Ongoing (https://doi.org/10.48670/moi-00148). Environmental factors associated with this article can be accessed at https://doi.org/10.6084/m9.figshare.25772259.v3. The sequence data have been submitted to the GenBank databases (https://www.ncbi.nlm.nih.gov/) under accession number PRJNA1127518 and PRJNA1005344.

## SUPPLEMENTAL MATERIAL

Supplemental figures and tables spectrum.01020-25-s0001.pdf Figures S1 and S12; Tables S1 to S7.

ASM does not own the copyrights to Supplemental Material that may be linked to, or accessed through, an article. The authors have granted ASM a non-exclusive, world-wide license to publish the Supplemental Material files. Please contact the corresponding author directly for reuse.

## References

[1] Bardgett RD, Freeman C, Ostle NJ. 2008. Microbial contributions to climate change through carbon cycle feedbacks. ISME J 2:805–814. doi:10.1038/ismej.2008.58 18615117

[2] Jackson R, Gabric A. 2022. Climate change impacts on the marine cycling of biogenic sulfur: a review. Microorganisms 10:1581. doi:10.3390/microorganisms10081581 36013999 PMC9412504

[3] Moran MA. 2015. The global ocean microbiome. Science 350:aac8455. doi:10.1126/science.aac8455 26659059

[4] Azam F, Malfatti F. 2007. Microbial structuring of marine ecosystems. Nat Rev Microbiol 5:782–791. doi:10.1038/nrmicro1747 17853906

[5] Banerjee S, Schlaeppi K, van der Heijden MGA. 2018. Keystone taxa as drivers of microbiome structure and functioning. Nat Rev Microbiol 16:567–576. doi:10.1038/s41579-018-0024-1 29789680

[6] Li C, Wang L, Ji S, Chang M, Wang L, Gan Y, Liu J. 2021. The ecology of the plastisphere: microbial composition, function, assembly, and network in the freshwater and seawater ecosystems. Water Res 202:117428. doi:10.1016/j.watres.2021.117428 34303166

[7] Steele JA, Countway PD, Xia L, Vigil PD, Beman JM, Kim DY, Chow C-ET, Sachdeva R, Jones AC, Schwalbach MS, Rose JM, Hewson I, Patel A, Sun F, Caron DA, Fuhrman JA. 2011. Marine bacterial, archaeal and protistan association networks reveal ecological linkages. ISME J 5:1414–1425. doi:10.1038/ismej.2011.24 21430787 PMC3160682

[8] Zhao D, Shen F, Zeng J, Huang R, Yu Z, Wu QL. 2016. Network analysis reveals seasonal variation of co-occurrence correlations between Cyanobacteria and other bacterioplankton. Sci Total Environ 573:817–825. doi:10.1016/j.scitotenv.2016.08.150 27595939

[9] Doney SC, Ruckelshaus M, Emmett Duffy J, Barry JP, Chan F, English CA, Galindo HM, Grebmeier JM, Hollowed AB, Knowlton N, Polovina J, Rabalais NN, Sydeman WJ, Talley LD. 2012. Climate change impacts on marine ecosystems. Annu Rev Mar Sci 4:11–37. doi:10.1146/annurev-marine-041911-111611 22457967

[10] Brum JR, Ignacio-Espinoza JC, Roux S, Doulcier G, Acinas SG, Alberti A, Chaffron S, Cruaud C, de Vargas C, Gasol JM, et al.. 2015. Patterns and ecological drivers of ocean viral communities. Science 348:1261498. doi:10.1126/science.1261498 25999515

[11] Gilbert JA, Steele JA, Caporaso JG, Steinbrück L, Reeder J, Temperton B, Huse S, McHardy AC, Knight R, Joint I, Somerfield P, Fuhrman JA, Field D. 2012. Defining seasonal marine microbial community dynamics. ISME J 6:298–308. doi:10.1038/ismej.2011.107 21850055 PMC3260500

[12] Livermore JA, Jones SE. 2015. Local–global overlap in diversity informs mechanisms of bacterial biogeography. ISME J 9:2413–2422. doi:10.1038/ismej.2015.51 25848869 PMC4611505

[13] Lochte K, Pfannkuche O. 1987. Cyclonic cold-core eddy in the eastern North Atlantic. II. Nutrients, phytoplankton and bacterioplankton. Mar Ecol Prog Ser 39:153–164. doi:10.3354/meps039153

[14] Mann KH, Lazier JRN. 2013. Dynamics of marine ecosystems: biological-physical interactions in the oceans. J Ecol 80:580. doi:10.1002/9781118687901

[15] Behrenfeld MJ, Boss ES. 2018. Student’s tutorial on bloom hypotheses in the context of phytoplankton annual cycles. Glob Chang Biol 24:55–77. doi:10.1111/gcb.13858 28787760 PMC5763361

[16] Müller AL, Kjeldsen KU, Rattei T, Pester M, Loy A. 2015. Phylogenetic and environmental diversity of DsrAB-type dissimilatory (bi)sulfite reductases. ISME J 9:1152–1165. doi:10.1038/ismej.2014.208 25343514 PMC4351914

[17] Sunagawa S, Coelho LP, Chaffron S, Kultima JR, Labadie K, Salazar G, Djahanschiri B, Zeller G, Mende DR, Alberti A, et al.. 2015. Structure and function of the global ocean microbiome. Science 348:1261359. doi:10.1126/science.1261359 25999513

[18] Mason OU, Scott NM, Gonzalez A, Robbins-Pianka A, Bælum J, Kimbrel J, Bouskill NJ, Prestat E, Borglin S, Joyner DC, Fortney JL, Jurelevicius D, Stringfellow WT, Alvarez-Cohen L, Hazen TC, Knight R, Gilbert JA, Jansson JK. 2014. Metagenomics reveals sediment microbial community response to Deepwater Horizon oil spill. ISME J 8:1464–1475. doi:10.1038/ismej.2013.254 24451203 PMC4069396

[19] Fuhrman JA, Cram JA, Needham DM. 2015. Marine microbial community dynamics and their ecological interpretation. Nat Rev Microbiol 13:133–146. doi:10.1038/nrmicro3417 25659323

[20] Hu J, Kawamura H, Hong H, Qi Y. 2000. A review on the currents in the South China Sea: seasonal circulation, South China Sea warm current and Kuroshio intrusion. J Oceanogr 56:607–624. doi:10.1023/A:1011117531252

[21] Muller-Karger FE, McClain CR, Richardson PL. 1988. The dispersal of the Amazon’s water. Nature 333:56–59. doi:10.1038/333056a0

[22] Wong GTF, Ku T-L, Mulholland M, Tseng C-M, Wang D-P. 2007. The SouthEast Asian Time-series Study (SEATS) and the biogeochemistry of the South China Sea—an overview. Deep Sea Res Part II Top Stud Oceanogr 54:1434–1447. doi:10.1016/j.dsr2.2007.05.012

[23] Ning X, Chai F, Xue H, Cai Y, Liu C, Shi J. 2004. Physical‐biological oceanographic coupling influencing phytoplankton and primary production in the South China Sea. J Geophys Res 109. doi:10.1029/2004JC002365

[24] Chen G, Hou Y, Chu X. 2011. Mesoscale eddies in the South China Sea: mean properties, spatiotemporal variability, and impact on thermohaline structure. J Geophys Res 116. doi:10.1029/2010JC006716

[25] Wang G, Su J, Chu PC. 2003. Mesoscale eddies in the South China Sea observed with altimeter data. Geophys Res Lett 30:2121. doi:10.1029/2003GL018532

[26] Hu J, Wang XH. 2016. Progress on upwelling studies in the China seas. Rev Geophys 54:653–673. doi:10.1002/2015RG000505

[27] Wang S, Sen K, He Y, Bai M, Wang G. 2022. Riverine inputs impact the diversity and population structure of heterotrophic fungus-like protists and bacterioplankton in the coastal waters of the South China Sea. Water (Basel) 14:1580. doi:10.3390/w14101580

[28] Li J, Jiang X, Li G, Jing Z, Zhou L, Ke Z, Tan Y. 2017. Distribution of picoplankton in the northeastern South China Sea with special reference to the effects of the Kuroshio intrusion and the associated mesoscale eddies. Sci Total Environ 589:1–10. doi:10.1016/j.scitotenv.2017.02.208 28273592

[29] Xu J, Li X, Shi Z, Li R, Li Q. 2018. Bacterial carbon cycling in the river plume in the northern South China Sea during summer. JGR Oceans 123:8106–8121. doi:10.1029/2018JC014277

[30] Zhou S, Liu J, Yao P, Fu L, Yang Z, Zhang Y, Du R, Jia C, Chen L, Liang J, Wang X, Shi X, Zhang X-H, Yu M. 2023. Unique bacterial communities and lifestyles in deep ocean blue holes: insights from the Yongle Blue Hole (South China Sea). Front Mar Sci 10:1086117. doi:10.3389/fmars.2023.1086117

[31] Zhu Y, Sun J, Wang Y, Li S, Xu T, Wei Z, Qu T. 2019. Overview of the multi-layer circulation in the South China Sea. Prog Oceanogr 175:171–182. doi:10.1016/j.pocean.2019.04.001

[32] Wang Y, Zhang R, He Z, Van Nostrand JD, Zheng Q, Zhou J, Jiao N. 2017. Functional gene diversity and metabolic potential of the microbial community in an estuary-shelf environment. Front Microbiol 8:1153. doi:10.3389/fmicb.2017.01153 28680420 PMC5478683

[33] Amir A, McDonald D, Navas-Molina JA, Kopylova E, Morton JT, Zech Xu Z, Kightley EP, Thompson LR, Hyde ER, Gonzalez A, Knight R. 2017. Deblur rapidly resolves single-nucleotide community sequence patterns. mSystems 2:e00191-16. doi:10.1128/mSystems.00191-16 28289731 PMC5340863

[34] Han A, Dai M, Kao S-J, Gan J, Li Q, Wang L, Zhai W, Wang L. 2012. Nutrient dynamics and biological consumption in a large continental shelf system under the influence of both a river plume and coastal upwelling. Limnol Oceanogr 57:486–502. doi:10.4319/lo.2012.57.2.0486

[35] Holmes RM, Aminot A, Kérouel R, Hooker BA, Peterson BJ. 1999. A simple and precise method for measuring ammonium in marine and freshwater ecosystems. Can J Fish Aquat Sci 56:1801–1808. doi:10.1139/f99-128

[36] Zheng Q, Wang Y, Xie R, Lang AS, Liu Y, Lu J, Zhang X, Sun J, Suttle CA, Jiao N. 2018. Dynamics of heterotrophic bacterial assemblages within Synechococcus cultures. Appl Environ Microbiol 84:e01517-17. doi:10.1128/AEM.01517-17 29150500 PMC5772231

[37] Deng Y, Jiang YH, Yang Y, He Z, Luo F, Zhou J. 2012. Molecular ecological network analyses. BMC Bioinform 13:113. doi:10.1186/1471-2105-13-113 PMC3428680 22646978

[38] Csárdi G, Nepusz T. 2006. The igraph software package for complex network research. https://github.com/igraph/rigraph.

[39] Wen T, Xie P, Yang S, Niu G, Liu X, Ding Z, Xue C, Liu Y-X, Shen Q, Yuan J. 2022. ggClusterNet: an R package for microbiome network analysis and modularity-based multiple network layouts. Imeta 1:e32. doi:10.1002/imt2.32 38868720 PMC10989811

[40] Ning D, Yuan M, Wu L, Zhang Y, Guo X, Zhou X, Yang Y, Arkin AP, Firestone MK, Zhou J. 2020. A quantitative framework reveals ecological drivers of grassland microbial community assembly in response to warming. Nat Commun 11:4717. doi:10.1038/s41467-020-18560-z 32948774 PMC7501310

[41] Sexton JP, Montiel J, Shay JE, Stephens MR, Slatyer RA. 2017. Evolution of ecological niche breadth. Annu Rev Ecol Evol Syst 48:183–206. doi:10.1146/annurev-ecolsys-110316-023003

[42] Tripathi BM, Stegen JC, Kim M, Dong K, Adams JM, Lee YK. 2018. Soil pH mediates the balance between stochastic and deterministic assembly of bacteria. ISME J 12:1072–1083. doi:10.1038/s41396-018-0082-4 29515169 PMC5864241

[43] Wang K, Yan H, Peng X, Hu H, Zhang H, Hou D, Chen W, Qian P, Liu J, Cai J, Chai X, Zhang D. 2020. Community assembly of bacteria and archaea in coastal waters governed by contrasting mechanisms: a seasonal perspective. Mol Ecol 29:3762–3776. doi:10.1111/mec.15600 32812678

[44] Oksanen J, Blanchet FG, Kindt R, Legendre P, Minchin P, O’Hara B, Simpson G, Solymos P, Stevens H, Wagner H. 2015. vegan: community ecology package. R Package Version 22-1 2:1–2. http://CRAN.R-project.org/package=vegan.

[45] Dolnicar S, Grabler K, Mazanec JA, Woodside A, Crouch G, Oppermann M. 1998. A tale of three cities: perceptual charting for analyzing destination images. Citeseer. https://research.wu.ac.at/en/publications/a-tale-of-three-cities-perceptual-charting-for-analyzing-destinat-11.

[46] Wu W, Lu H-P, Sastri A, Yeh Y-C, Gong G-C, Chou W-C, Hsieh C-H. 2018. Contrasting the relative importance of species sorting and dispersal limitation in shaping marine bacterial versus protist communities. ISME J 12:485–494. doi:10.1038/ismej.2017.183 29125596 PMC5776463

[47] Douglas GM, Maffei VJ, Zaneveld JR, Yurgel SN, Brown JR, Taylor CM, Huttenhower C, Langille MGI. 2020. PICRUSt2 for prediction of metagenome functions. Nat Biotechnol 38:685–688. doi:10.1038/s41587-020-0548-6 32483366 PMC7365738

[48] Louca S, Parfrey LW, Doebeli M. 2016. Decoupling function and taxonomy in the global ocean microbiome. Science 353:1272–1277. doi:10.1126/science.aaf4507 27634532

[49] Xu S, Hu E, Cai Y, Xie Z, Luo X, Zhan L, Tang W, Wang Q, Liu B, Wang R, Xie W, Wu T, Xie L, Yu G. 2024. Using clusterProfiler to characterize multiomics data. Nat Protoc 19:3292–3320. doi:10.1038/s41596-024-01020-z 39019974

[50] Wickham H. 2016. ggplot2: elegant graphics for data analysis

[51] Kassambara A. 2020. ggpubr: “ggplot2” based publication ready plots (R package version 0.4.0). https://CRAN.R-project.org/package=ggpubr.

[52] Liaw A, Wiener M. 2002. Classification and regression by randomForest. R News 2:18–22.

[53] Milke F, Meyerjürgens J, Simon M. 2023. Ecological mechanisms and current systems shape the modular structure of the global oceans’ prokaryotic seascape. Nat Commun 14:6141. doi:10.1038/s41467-023-41909-z 37783696 PMC10545751

[54] Huber P, Metz S, Unrein F, Mayora G, Sarmento H, Devercelli M. 2020. Environmental heterogeneity determines the ecological processes that govern bacterial metacommunity assembly in a floodplain river system. ISME J 14:2951–2966. doi:10.1038/s41396-020-0723-2 32719401 PMC7784992

[55] Wilkins D, van Sebille E, Rintoul SR, Lauro FM, Cavicchioli R. 2013. Advection shapes southern ocean microbial assemblages independent of distance and environment effects. Nat Commun 4:2457. doi:10.1038/ncomms3457 24036630

[56] Hamdan LJ, Coffin RB, Sikaroodi M, Greinert J, Treude T, Gillevet PM. 2013. Ocean currents shape the microbiome of Arctic marine sediments. ISME J 7:685–696. doi:10.1038/ismej.2012.143 23190727 PMC3603395

[57] Doblin MA, van Sebille E. 2016. Drift in ocean currents impacts intergenerational microbial exposure to temperature. Proc Natl Acad Sci USA 113:5700–5705. doi:10.1073/pnas.1521093113 27140608 PMC4878470

[58] Xian W-D, Chen J, Zheng Z, Ding J, Xi Y, Zhang Y, Qu W, Tang C, Li C, Liu X, Li W, Wang J. 2024. Water masses influence the variation of microbial communities in the Yangtze River estuary and its adjacent waters. Front Microbiol 15:1367062. doi:10.3389/fmicb.2024.1367062 38572235 PMC10987813

[59] Tee HS, Waite D, Lear G, Handley KM. 2021. Microbial river-to-sea continuum: gradients in benthic and planktonic diversity, osmoregulation and nutrient cycling. Microbiome 9:190. doi:10.1186/s40168-021-01145-3 34544488 PMC8454136

[60] Hanson CA, Fuhrman JA, Horner-Devine MC, Martiny JBH. 2012. Beyond biogeographic patterns: processes shaping the microbial landscape. Nat Rev Microbiol 10:497–506. doi:10.1038/nrmicro2795 22580365

[61] Nelson CE, Wegley Kelly L, Haas AF. 2023. Microbial interactions with dissolved organic matter are central to coral reef ecosystem function and resilience. Annu Rev Mar Sci 15:431–460. doi:10.1146/annurev-marine-042121-080917 36100218

[62] Lin S, Gan J. 2024. Dynamics of tidal effects on coastal upwelling circulation over variable shelves in the northern South China Sea. J Geophys Res Oceans 129:e2024JC021193. doi:10.1029/2024JC021193

[63] Junger PC, Sarmento H, Giner CR, Mestre M, Sebastián M, Morán XAG, Arístegui J, Agustí S, Duarte CM, Acinas SG, Massana R, Gasol JM, Logares R. 2023. Global biogeography of the smallest plankton across ocean depths. Sci Adv 9:eadg9763. doi:10.1126/sciadv.adg9763 37939185 PMC10631730

[64] Galand PE, Potvin M, Casamayor EO, Lovejoy C. 2010. Hydrography shapes bacterial biogeography of the deep Arctic ocean. ISME J 4:564–576. doi:10.1038/ismej.2009.134 20010630

[65] Sun P, Wang Y, Huang X, Huang B, Wang L. 2022. Water masses and their associated temperature and cross-domain biotic factors co-shape upwelling microbial communities. Water Res 215:118274. doi:10.1016/j.watres.2022.118274 35298994

[66] Agogué H, Lamy D, Neal PR, Sogin ML, Herndl GJ. 2011. Water mass-specificity of bacterial communities in the North Atlantic revealed by massively parallel sequencing. Mol Ecol 20:258–274. doi:10.1111/j.1365-294X.2010.04932.x 21143328 PMC3057482

[67] Gómez-Letona M, Arístegui J, Hernández-Hernández N, Álvarez-Salgado XA, Álvarez M, Delgadillo E, Pérez-Lorenzo M, Teira E, Hernández-León S, Sebastián M. 2022. Deep ocean prokaryotes and fluorescent dissolved organic matter reflect the history of the water masses across the Atlantic ocean. Prog Oceanogr 205:102819. doi:10.1016/j.pocean.2022.102819

[68] Martínez–Pérez AM, Catalá TS, Nieto–Cid M, Otero J, Álvarez M, Emelianov M, Reche I, Álvarez–Salgado XA, Arístegui J. 2019. Dissolved organic matter (DOM) in the open Mediterranean Sea. II: basin–wide distribution and drivers of fluorescent DOM. Prog Oceanogr 170:93–106. doi:10.1016/j.pocean.2018.10.019

[69] Dong H-P, Wang D-Z, Xie Z-X, Dai M-H, Hong H-S. 2013. Metaproteomic characterization of high molecular weight dissolved organic matter in surface seawaters in the South China Sea. Geochim Cosmochim Acta 109:51–61. doi:10.1016/j.gca.2013.01.041

[70] Wu K, Dai M, Chen J, Meng F, Li X, Liu Z, Du C, Gan J. 2015. Dissolved organic carbon in the South China Sea and its exchange with the Western Pacific Ocean. Deep Sea Res II Top Stud Oceanogr 122:41–51. doi:10.1016/j.dsr2.2015.06.013

[71] Raes EJ, Bodrossy L, van de Kamp J, Bissett A, Ostrowski M, Brown MV, Sow SLS, Sloyan B, Waite AM. 2018. Oceanographic boundaries constrain microbial diversity gradients in the South Pacific ocean. Proc Natl Acad Sci USA 115:E8266–E8275. doi:10.1073/pnas.1719335115 30108147 PMC6126737

[72] Catalá TS, Reche I, Álvarez M, Khatiwala S, Guallart EF, Benítez‐Barrios VM, Fuentes‐Lema A, Romera‐Castillo C, Nieto‐Cid M, Pelejero C, Fraile‐Nuez E, Ortega‐Retuerta E, Marrasé C, Álvarez‐Salgado XA. 2015. Water mass age and aging driving chromophoric dissolved organic matter in the dark global ocean. Global Biogeochem Cycles 29:917–934. doi:10.1002/2014GB005048

[73] Ke Z, Tan Y, Huang L, Zhang J, Lian S. 2012. Relationship between phytoplankton composition and environmental factors in the surface waters of southern South China Sea in early summer of 2009. Acta Oceanol Sin 31:109–119. doi:10.1007/s13131-012-0211-2

[74] de Santana CO, Spealman P, Melo VMM, Gresham D, de Jesus TB, Chinalia FA. 2021. Effects of tidal influence on the structure and function of prokaryotic communities in the sediments of a pristine Brazilian mangrove. Biogeosciences 18:2259–2273. doi:10.5194/bg-18-2259-2021

[75] Maturana-Martínez C, Iriarte JL, Ha S-Y, Lee B, Ahn I-Y, Vernet M, Cape M, Fernández C, González HE, Galand PE. 2022. Biogeography of southern ocean active prokaryotic communities over a large spatial scale. Front Microbiol 13:862812. doi:10.3389/fmicb.2022.862812 35592001 PMC9111744

[76] Kong J, Wang L, Lin C, Kuang F, Zhou X, Laws EA, Sun P, Huang H, Huang B. 2022. Contrasting community assembly mechanisms underlie similar biogeographic patterns of surface microbiota in the tropical North Pacific ocean. Microbiol Spectr 10:e00798-21. doi:10.1128/spectrum.00798-21 35019678 PMC8754141

[77] Stegen JC, Lin X, Fredrickson JK, Chen X, Kennedy DW, Murray CJ, Rockhold ML, Konopka A. 2013. Quantifying community assembly processes and identifying features that impose them. ISME J 7:2069–2079. doi:10.1038/ismej.2013.93 23739053 PMC3806266

[78] Kirchman DL. 2016. Growth rates of microbes in the oceans. Ann Rev Mar Sci 8:285–309. doi:10.1146/annurev-marine-122414-033938 26195108

[79] Legendre P, Mi X, Ren H, Ma K, Yu M, Sun I-F, He F. 2009. Partitioning beta diversity in a subtropical broad‐leaved forest of China. Ecol 90:663–674. doi:10.1890/07-1880.1 19341137

[80] Shi Z, Ma L, Wang Y, Liu J. 2023. Abundant and rare bacteria in anthropogenic estuary: community co-occurrence and assembly patterns. Ecol Indic 146:109820. doi:10.1016/j.ecolind.2022.109820

[81] Isabwe A, Yang JR, Wang Y, Wilkinson DM, Graham EB, Chen H, Yang J. 2022. Riverine bacterioplankton and phytoplankton assembly along an environmental gradient induced by urbanization. Limnol Oceanogr 67:1943–1958. doi:10.1002/lno.12179

[82] Tu Q, Yan Q, Deng Y, Michaletz ST, Buzzard V, Weiser MD, Waide R, Ning D, Wu L, He Z, Zhou J. 2020. Biogeographic patterns of microbial co-occurrence ecological networks in six American forests. Soil Biol Biochem 148:107897. doi:10.1016/j.soilbio.2020.107897

[83] Milke F, Wagner-Doebler I, Wienhausen G, Simon M. 2022. Selection, drift and community interactions shape microbial biogeographic patterns in the Pacific ocean. ISME J 16:2653–2665. doi:10.1038/s41396-022-01318-4 36115923 PMC9666467

[84] Yuan MM, Guo X, Wu L, Zhang Y, Xiao N, Ning D, Shi Z, Zhou X, Wu L, Yang Y, Tiedje JM, Zhou J. 2021. Climate warming enhances microbial network complexity and stability. Nat Clim Chang 11:343–348. doi:10.1038/s41558-021-00989-9

[85] Hughes AR, Inouye BD, Johnson MTJ, Underwood N, Vellend M. 2008. Ecological consequences of genetic diversity. Ecol Lett 11:609–623. doi:10.1111/j.1461-0248.2008.01179.x 18400018

[86] Cram JA, Xia LC, Needham DM, Sachdeva R, Sun F, Fuhrman JA. 2015. Cross-depth analysis of marine bacterial networks suggests downward propagation of temporal changes. ISME J 9:2573–2586. doi:10.1038/ismej.2015.76 25989373 PMC4817623

[87] Jiang Y, Sun B, Li H, Liu M, Chen L, Zhou S. 2015. Aggregate-related changes in network patterns of nematodes and ammonia oxidizers in an acidic soil. Soil Biol Biochem 88:101–109. doi:10.1016/j.soilbio.2015.05.013

[88] Chen G, Wang W, Zhang Y, Liu Y, Gu X, Shi X, Wang M. 2020. Abundant and rare species may invoke different assembly processes in response to climate extremes: implications for biodiversity conservation. Ecol Indic 117:106716. doi:10.1016/j.ecolind.2020.106716

[89] Liang Y, Xiao X, Nuccio EE, Yuan M, Zhang N, Xue K, Cohan FM, Zhou J, Sun B. 2020. Differentiation strategies of soil rare and abundant microbial taxa in response to changing climatic regimes. Environ Microbiol 22:1327–1340. doi:10.1111/1462-2920.14945 32067386

[90] Xiong C, He J-Z, Singh BK, Zhu Y-G, Wang J-T, Li P-P, Zhang Q-B, Han L-L, Shen J-P, Ge A-H, Wu C-F, Zhang L-M. 2021. Rare taxa maintain the stability of crop mycobiomes and ecosystem functions. Environ Microbiol 23:1907–1924. doi:10.1111/1462-2920.15262 32996254

[91] Wang C, Guo L, Cai ZJ, Chen J, Shen RF. 2024. Different contributions of rare microbes to driving soil nitrogen cycles in acidic soils under manure fertilization. Appl Soil Ecol 196:105281. doi:10.1016/j.apsoil.2024.105281

[92] Röttjers L, Faust K. 2018. From hairballs to hypotheses-biological insights from microbial networks. FEMS Microbiol Rev 42:761–780. doi:10.1093/femsre/fuy030 30085090 PMC6199531

[93] Modlmeier AP, Keiser CN, Watters JV, Sih A, Pruitt JN. 2014. The keystone individual concept: an ecological and evolutionary overview. Anim Behav 89:53–62. doi:10.1016/j.anbehav.2013.12.020

[94] Ning D, Wang Y, Fan Y, Wang J, Van Nostrand JD, Wu L, Zhang P, Curtis DJ, Tian R, Lui L, Hazen TC, Alm EJ, Fields MW, Poole F, Adams MWW, Chakraborty R, Stahl DA, Adams PD, Arkin AP, He Z, Zhou J. 2024. Environmental stress mediates groundwater microbial community assembly. Nat Microbiol 9:490–501. doi:10.1038/s41564-023-01573-x 38212658

[95] Chen Z, Sun J, Chen D, Wang S, Yu H, Chen H, Wang M. 2021. Effects of ocean currents in the Western Pacific ocean on net-phytoplankton community compositions. Diversity (Basel) 13:428. doi:10.3390/d13090428

