# Supplementary material for: Ocean currents and environmental gradients shape prokaryotic community structure and function in the South China Sea
Source: Microbiol Spectr. 2025 Sep 24;13(11):e01020-25. doi: 10.1128/spectrum.01020-25 (PMC12584625; doi:10.1128/spectrum.01020-25)
Supplement: Supplemental figures and tables — Figures S1 and S12; Tables S1 to S7. [file spectrum.01020-25-s0001.pdf]

Supplementary information for Ocean Currents and Environmental Gradients Shape Prokaryotic Community Structure and Function in the South China Sea

Yu Wang[1,2,3]†*, Jinxin Xu[1,2]†, Yanting Liu[1,2], Lu Liu[1,2], Shicong Xiao[1,2,4], Xiaomeng Wang[1,2,5], Jiandong, Zhang[6], Sijun Huang[6], Qiang Zheng[1,2]*

[1]State Key Laboratory for Marine Environmental Science, Institute of Marine Microbes and Ecospheres, College of Ocean and Earth Sciences, Xiamen University, Xiamen 361005, People's Republic of China

[2]Fujian Key Laboratory of Marine Carbon Sequestration, Xiamen University, Xiamen 361005, People's Republic of China

[3]College of Environment and Ecology, Xiamen University, Xiamen 361005, People's Republic of China

[4]Jiangsu Institute of Marine Resources Development, Jiangsu Ocean University, Lianyungang 222005, People's Republic of China

[5]Department of Ocean Science and Engineering, Southern University of Science and Technology, Shenzhen 518055, People's Republic of China

[6]CAS Key Laboratory of Tropical Marine Bio-resources and Ecology, South China Sea Institute of Oceanology, Chinese Academy of Sciences, Guangzhou 510301, People's Republic of China

*Authors for correspondence:

Yu Wang: wangyu@xmu.edu.cn

Qiang Zheng: zhengqiang@xmu.edu.cn

†These authors contributed equally to this work.

Supporting tables

Table S1. The average proportion of the top five taxonomic groups of prokaryotic communities in different water masses at the Class level.

Class	VWEB	VCEB	QDB	PRMB	LWEB
Cyanobacteriia	28.3±7.4%	27.7±10.3%	28.7±10.6%	34.1±11.5%	33.1±21.7%
Alphaproteobacteria	33.2±7.0%	35.3±8.4%	28.5±7.6%	22.6±7.6%	16.6±11.1%
Acidimicrobiia	9.6±3.6%	8.6±3.5%	13.3±4.2%	14.9±10.4%	21.6±17.3%
Bacteroidia	11.4±3.9%	11.6±5.2%	11.0±1.8%	10.6±3.1%	12.2±3.6%
Gammaproteobacteria	7.9±1.6%	7.9±2.2%	8.0±4.9%	7.0±4.1%	4.8±5.6%

Table S2. Abundance of microbial communities in each water mass in the South China Sea

Water mass[1]	Microeukaryotes (cells/mL)		Synechococcus (10^4 cells/mL)		Prochlorococcus (10^4 cells/mL)		Bacteria (10^5 cells/mL)	
	Raw[2]	Ln-transformed	Raw	Ln-transformed	Raw	Ln-transformed	Raw	Ln-transformed
VWEB	814±286	6.65±0.34	2.45±3.01	9.60±0.99	17.13±4.63	12.05±0.29	11.16±4.05	13.92±0.36
VCEB	1883±966	7.40±0.58	4.85±4.01	10.50±0.76	15.45±6.07	11.95±0.49	10.84±2.77	13.9±0.26
QDB	377±296	5.73±0.64	0.96±1.89	7.91±1.78	1.67±2.36	9.72±2.54	7.81±8.53	13.57±0.65
PRMB	815±1733	5.85±1.10	2.21±4.69	8.77±1.50	2.48±2.58	10.12±1.53	6.46±2.90	13.38±0.47
LWEB	165±95.1	4.85±0.93	0.64±0.54	8.27±1.27	1.82±1.18	9.81±1.76	7.05±2.31	13.47±0.27

[1] VWEB: Vietnam Warm Eddy Basin; VCEB: Vietnam Cold Eddy Basin; QDB: Qiongdong Basin; PRMB: Pearl River Mouth Basin; LWEB: Luzon Warm Eddy Basin.

[2] Mean ± s.d.

Table S3. Difference between water masses based on Bray-Curtis dissimilarity tested by PEMANOVA

	VCEB		QDB		PRMB		LWEB	
	R^2	p	R^2	p	R^2	p	R^2	p
VWEB	0.18	0.002	0.12	0.005	0.13	0.007	0.20	0.004
VCEB			0.24	0.001	0.18	0.002	0.24	0.001
QDB					0.05	0.121	0.11	0.016
PRMB							0.08	0.069

Table S4. The topological properties of co-occurrence networks of prokaryotic microbial in the South China Sea

	SCS	VWEB	VCEB	QDB	PRMB	LWEB
No. Edges (*L*)	1,712	447	197	476	543	1,195
No. Positive edges (L_p)	1,549	406	185	454	528	1,178
No. Negative edges (L_n)	163	41	12	22	15	17
No. Nodes (*n*)	353	356	184	233	308	286
Connectance (Con)	0.028	0.007	0.011	0.013	0.011	0.029
Average degree (Ave. *K*)	9.7	2.444	2.073	3.353	3.448	8.357
Average path distance	2.296	4.298	1.413	2.637	3.372	3.468
Diameter (*D*)	5.902	12.901	4.45	8.319	11.242	12.949
Average clustering coefficient (Ave. *CC*)	0.462	0.617	0.732	0.675	0.658	0.592
No. Module	6	90	63	60	71	33
Centralization of degree (Centra. of degree)	0.117	0.022	0.038	0.047	0.041	0.086
Centralization of betweenness (Centra. of betweenness)	0.091	0.017	0.001	0.018	0.014	0.048
Centralization of closeness (Centra. of closeness)	1.391	0.579	0.24	0.479	0.563	1.006
Relative modularity (RM)	1.06	0.523	0.413	0.575	0.851	1.351

Table S5. Mantel and partial mantel test of Bray-Curtis and Sørensen dissimilarity of prokaryotic communities with environmental distance and geographic distances.

	Bray-Curtis dissimilarity		Sørensen dissimilarity	
	Mantel's r	p	Mantel's r	p
Environmental distance*	0.29	0.001	0.42	0.001
Geographic distance	0.21	0.001	0.43	0.001
Environmental distance | Geographic distance**	0.25	0.001	0.38	0.001
Geographic distance | Environmental distance	0.16	0.001	0.38	0.001

*Environmental distance based on scaled temperature, salinity, NO_x, NH_4, PO_4 and SiO_3 with Euclidean distance.

**Partial mantel test, the distance on the right of vertical line is the partial distance.

Table S6. R^2 of pairwise PERMANOVA analysis between water masses based on PICRUSt2 prediction.

	VWEB	VCEB	QDB	PRMB
VCEB	0.05			
QDB	0.11*	0.17***		
PRMB	0.11*	0.17***	0.05	
LWEB	0.20***	0.25***	0.14**	0.07

* p value < 0.05; ** p value < 0.01; *** p value < 0.001.

Table S7. Pearson correlation of modified stochasticity ratio (MST) with properties of subnetworks.

	r	p
L	-0.80	0.10
L_p	-0.82	0.09
L_n	0.62	0.26
n	0.03	0.96
Con	-0.87	0.06
Ave. K	-0.86	0.06
Ave. path distance	-0.02	0.98
D	-0.29	0.64
Ave. CC	0.38	0.53
No. Module	0.82	0.09
Centra. Of degree	-0.88	0.05
Centra. Of betweenness	-0.69	0.20
Centra. Of closeness	-0.69	0.20
RM	-0.91	0.03

Supporting figures

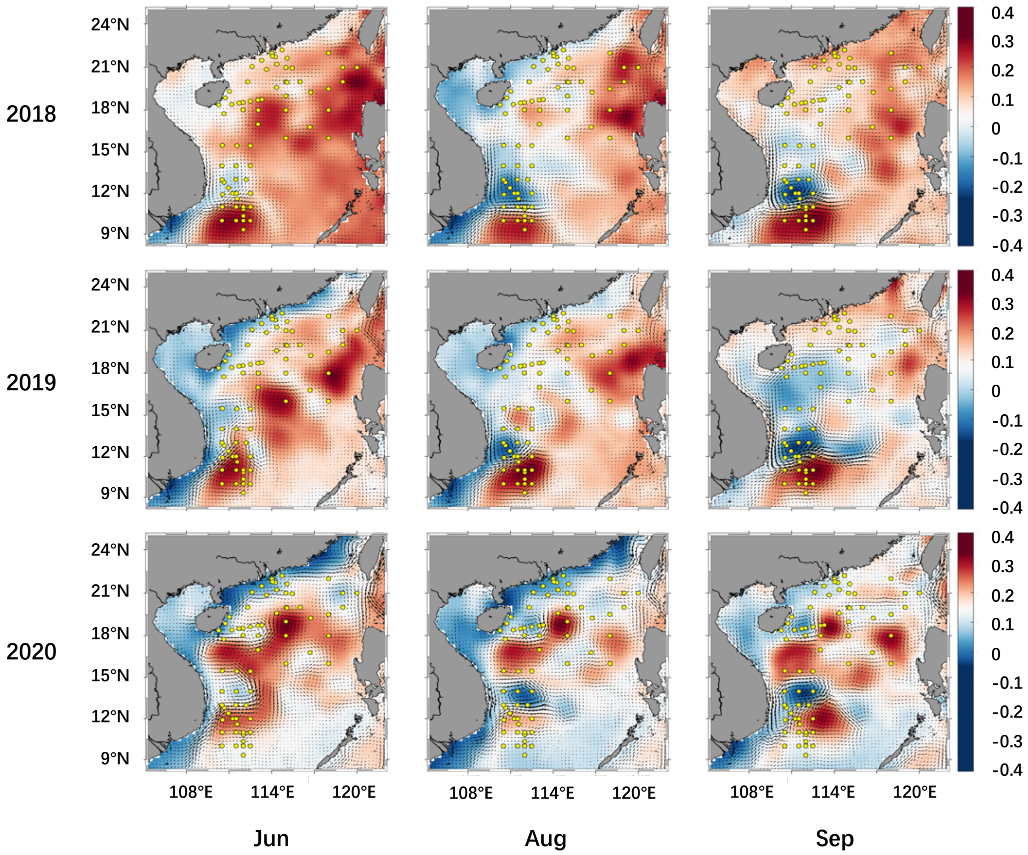

Figure S1. Monthly mean surface level anomaly and surface geostrophic current from July to September in 2018-2020.

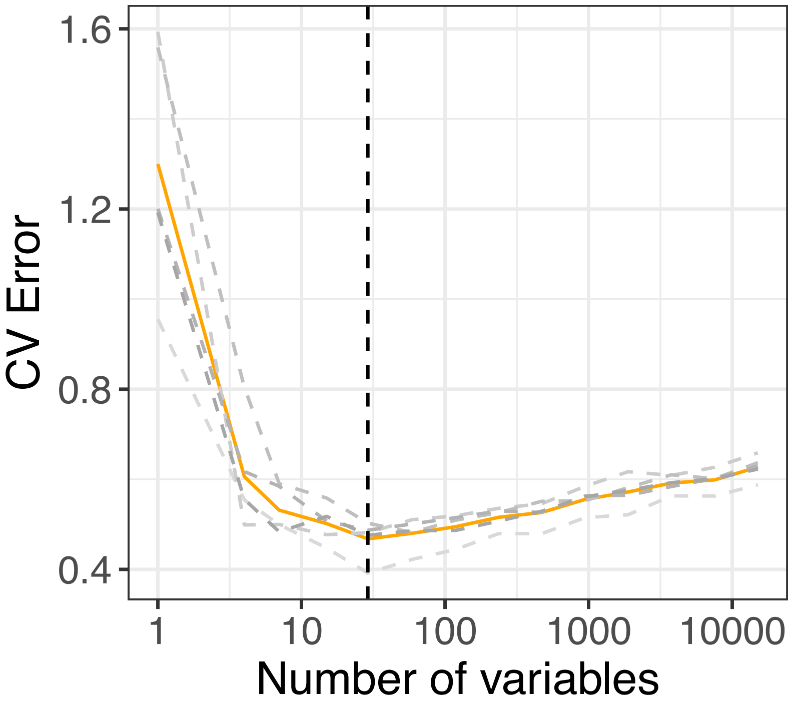

Figure S2. Cross-validation (CV) error of the random forest model with 10-fold cross validation. Orange line indicates the original result, while the gray dashed lines indicate five replicates.

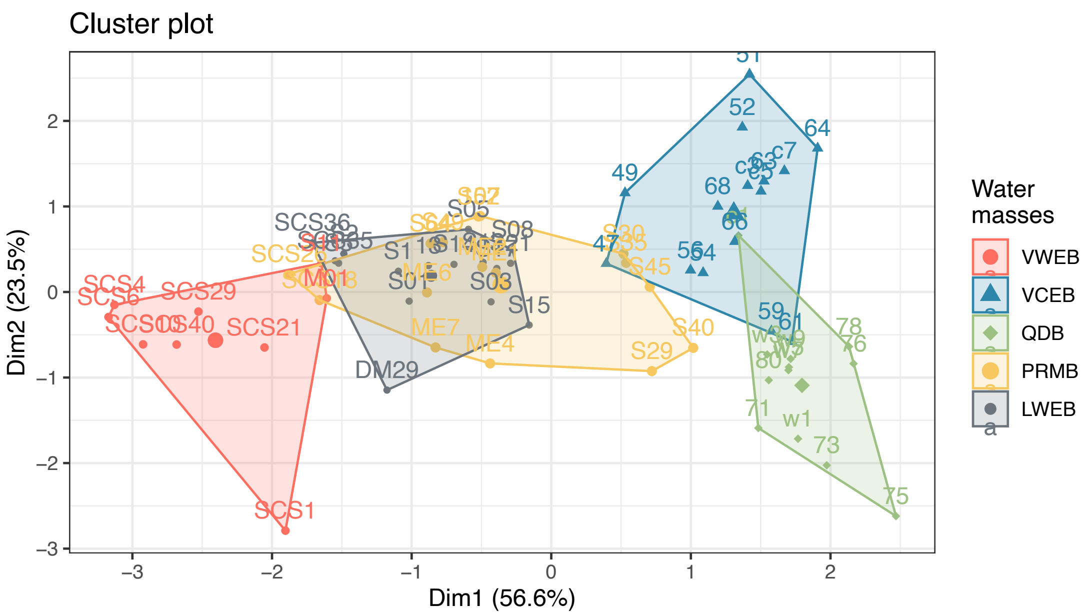

Figure S3. The fuzzy clustering of the water masses in the South China Sea based on the latitude, longitude, temperature and salinity. VWEB: Vietnam Warm Eddy Basin; VCEB: Vietnam Cold Eddy Basin; QDB: Qiongdong Basin; PRMB: the Pearl River Mouth Basin; LWEB: Luzon Warm Eddy Basin.

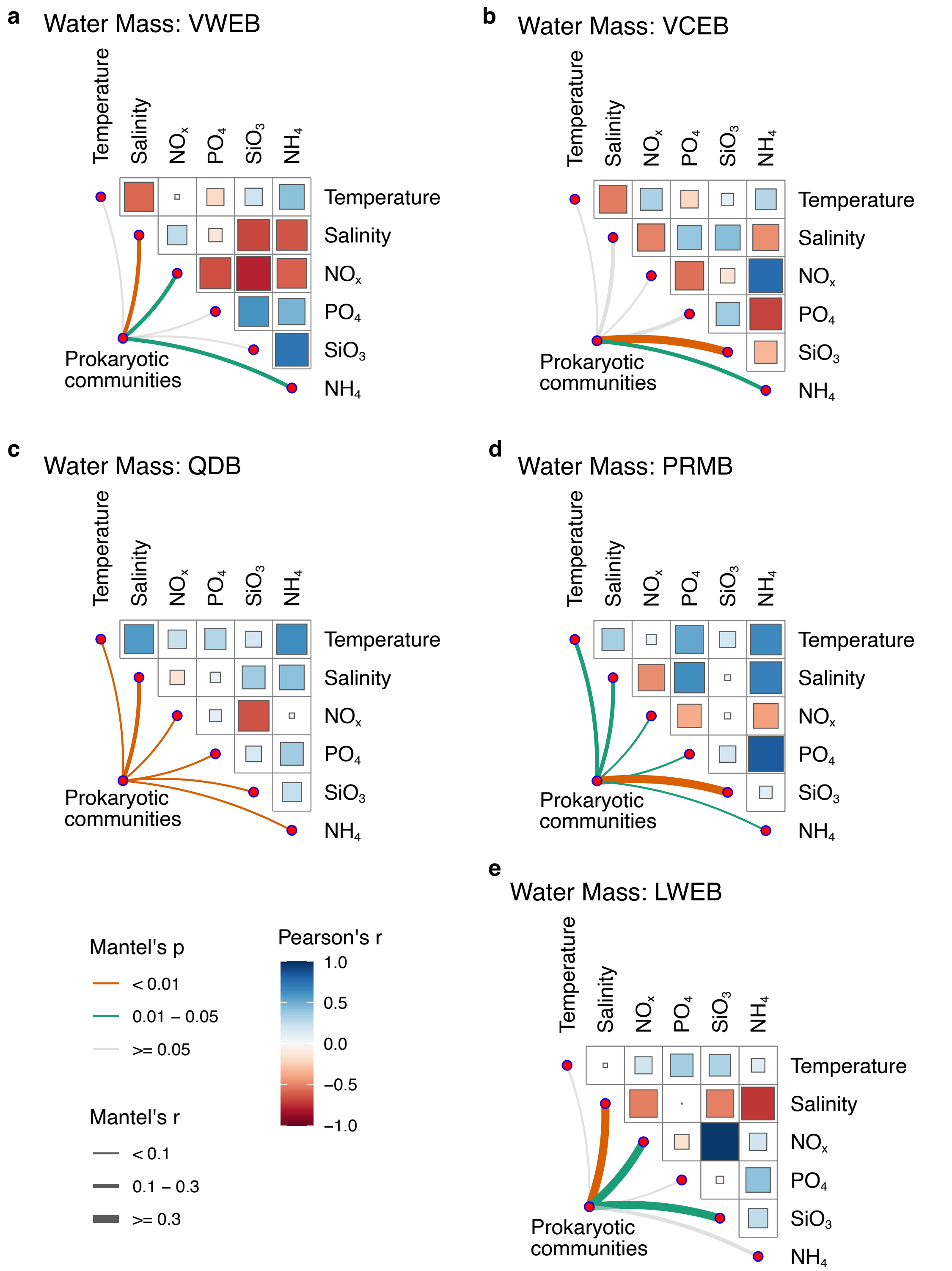

Figure S4. Environmental drivers of surface prokaryotic community composition within water masses. a VWEB: Vietnam Warm Eddy Basin; **b** VCEB: Vietnam Cold Eddy Basin; **c** QDB: Qiongdong Basin; **d** PRMB: the Pearl River Mouth Basin; **e** LWEB: Luzon Warm Eddy Basin. Pairwise comparisons of environmental factors are shown, with a color gradient denoting Pearson's

correlation coefficient. Taxonomic community composition was related to each environmental factor by partial (geographic distance–corrected) Mantel tests. Edge width corresponds to the Mantel's r statistic for the corresponding distance correlations, and edge color denotes the statistical significance based on 9,999 permutations.

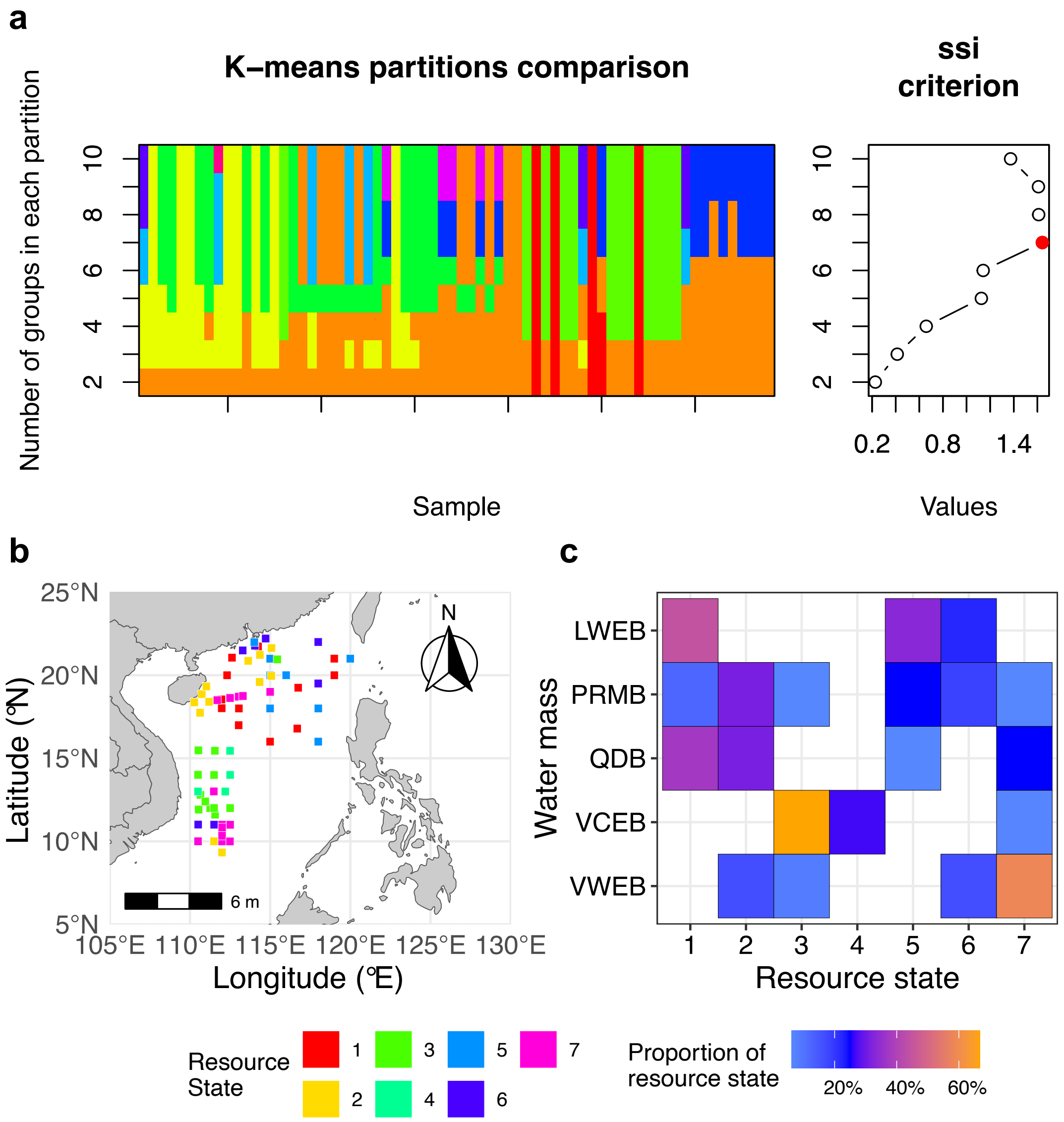

Figure S5. *K*-means partitioning of resource states and its spatial distribution. **a** *K*-means partitioning of resource states across sampling stations from south to north based on Euclidean distance of water environmental factors. The simple structure index (SSI) is used to determine the best *K* value (the optimal number of resource states). According to SSI criterion, the optimal number of resource states is seven. **b** Spatial distribution of the resource states determined by *K*-means partitioning based on water environmental conditions in the cruise of each season. The colors of the stations correspond to different resource states. **c** Proportion of resource states in each water mass (%).

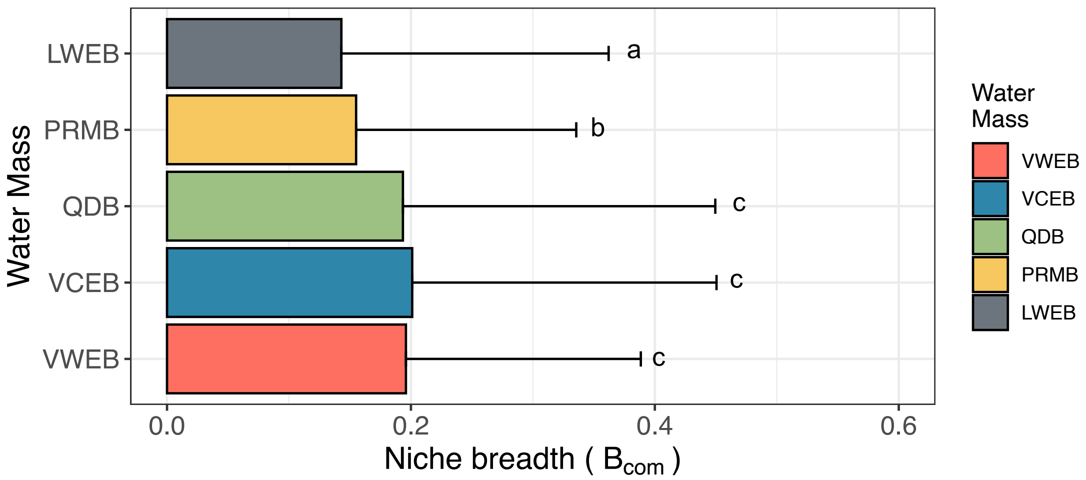

Figure S6. Standardized Levins' niche breadth of prokaryotes at the community level (B_{com}) in the different water masses. The significant difference in B_{com} between water masses is marked by letters. The same letter above the boxes is not significantly different from each other (multiple comparisons after Wilcoxon test, adjusted p value > 0.05), whereas two data sets with different letters are significantly different (adjusted p value < 0.001)

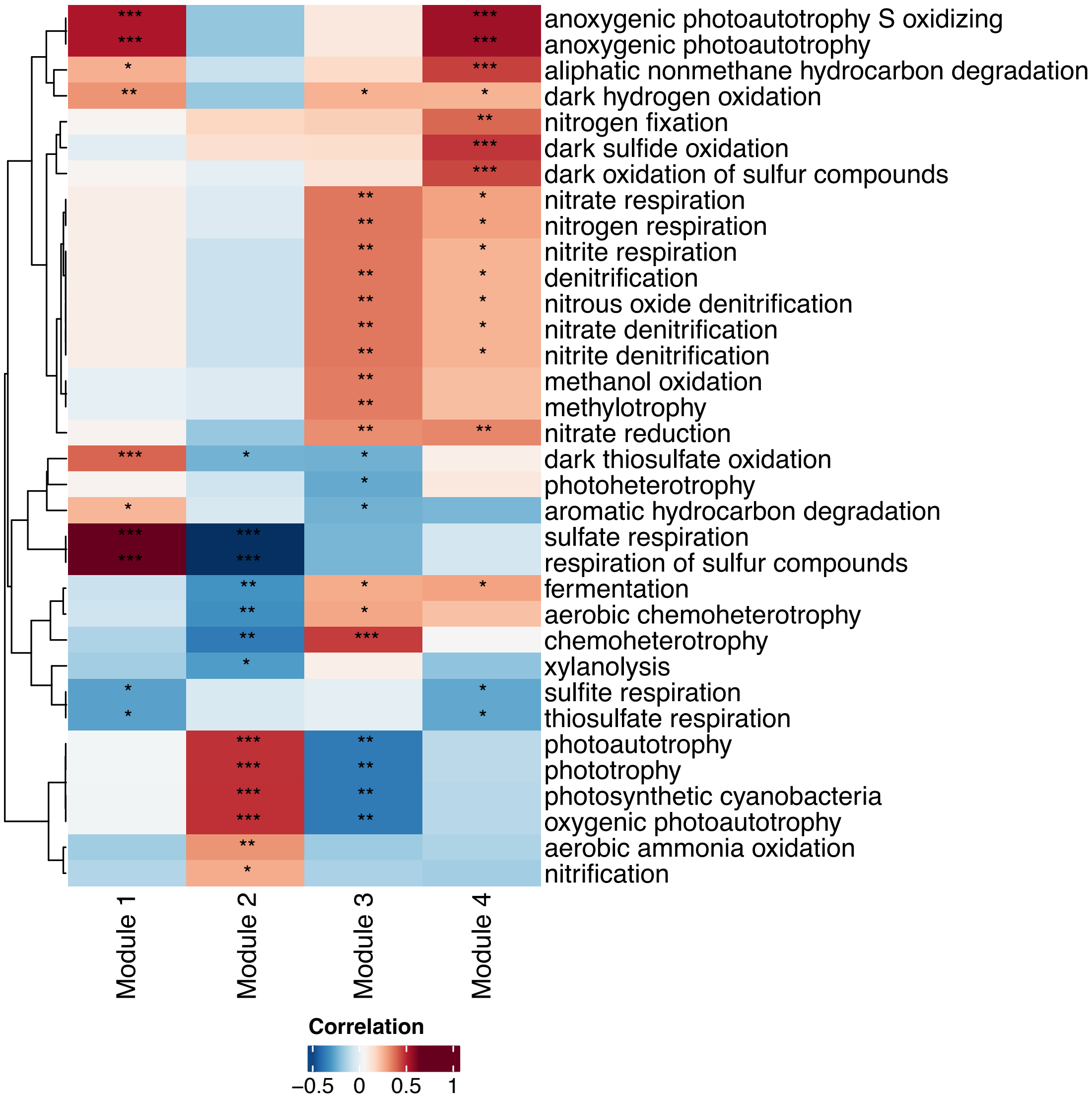

Figure S7. Spearman correlation between abundance of network modules and metabolism function predicted by FAPROTAX. *, p value < 0.05; **, p value < 0.01; ***, p value < 0.001.

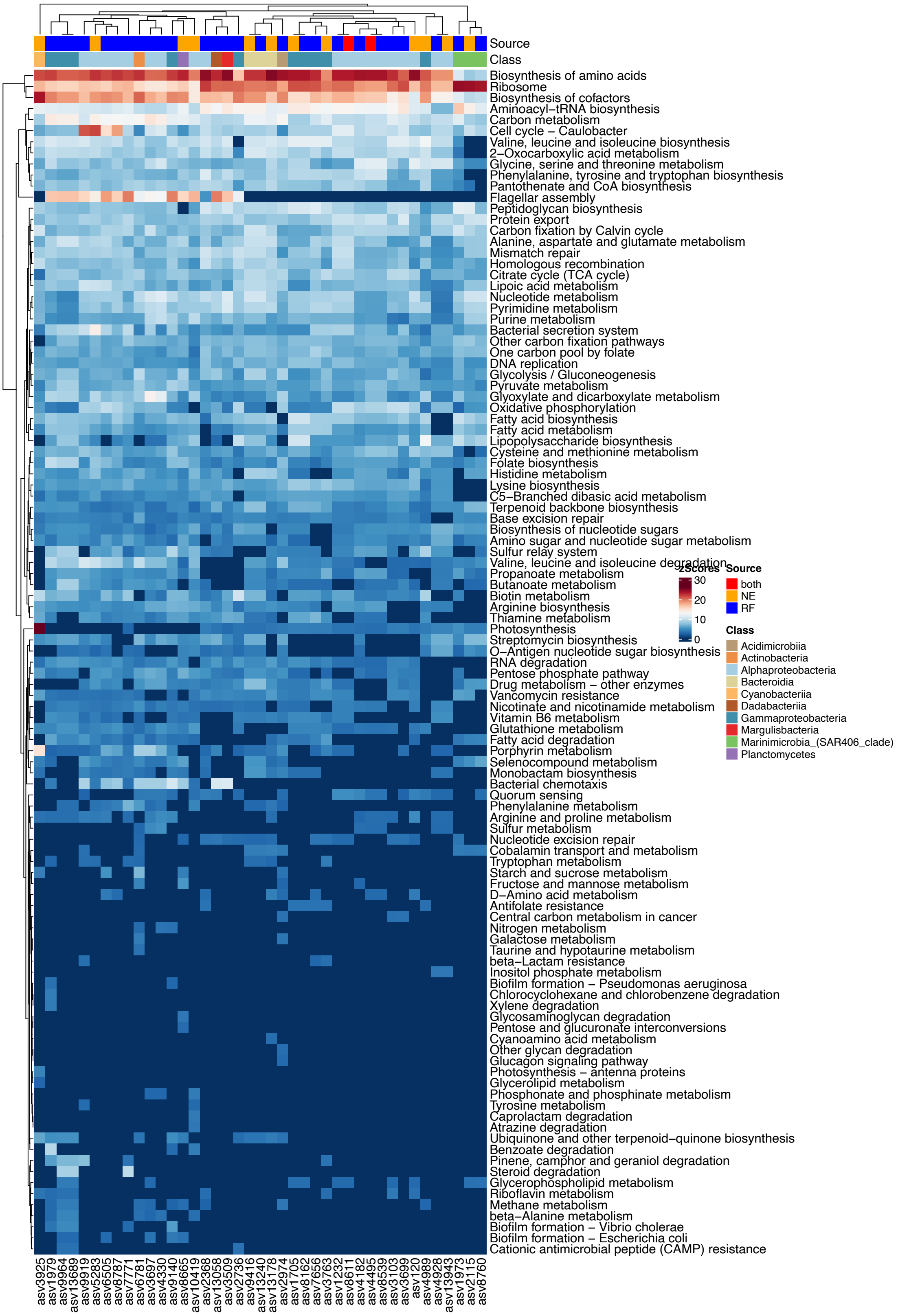

Figure S8. Metabolism enrichment of the prokaryotic communities based on the PICRUSt2 prediction (adjusted p < 0.05). Source indicates the ASV identified

by network (NE), random forest model (RF), or both. The Class color indicates the class of these ASVs.

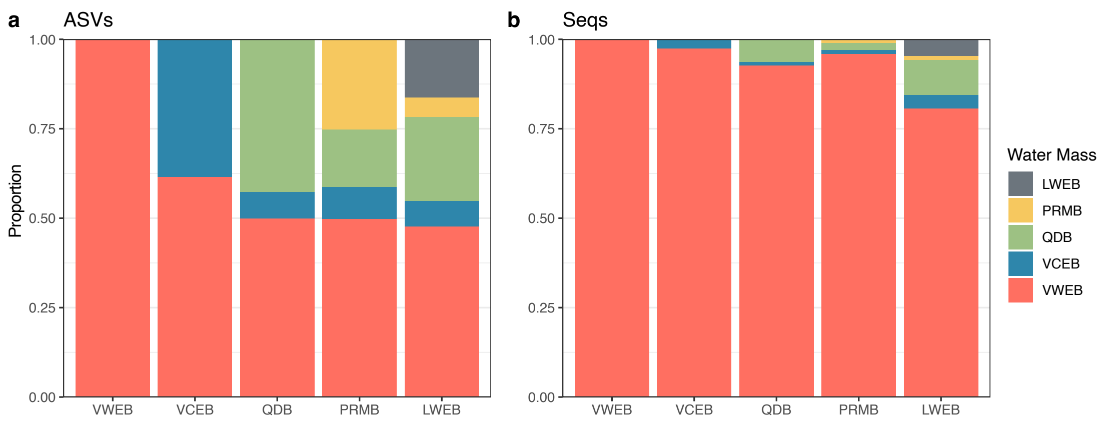

Figure S9. Contribution of ASVs in one water masses to other water masses. The proportion of ASVs categorized by where they were first detected, following the gradient of currents in the South China Sea. **a** Calculation was based on ASV number. **b** Calculation was based on sequence number. VWEB: Vietnam Warm Eddy Basin; VCEB: Vietnam Cold Eddy Basin; QDB: Qiongdong Basin; PRMB: the Pearl River Mouth Basin; LWEB: Luzon Warm Eddy Basin.

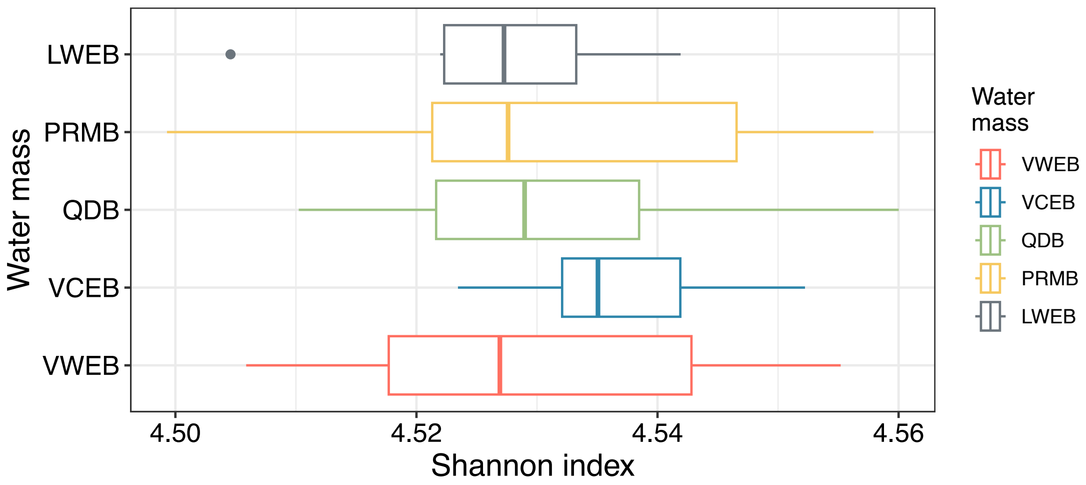

Figure S10. Shannon index based on the PICRUST2 prediction. ANOVA test F = 0.827, p = 0.51.

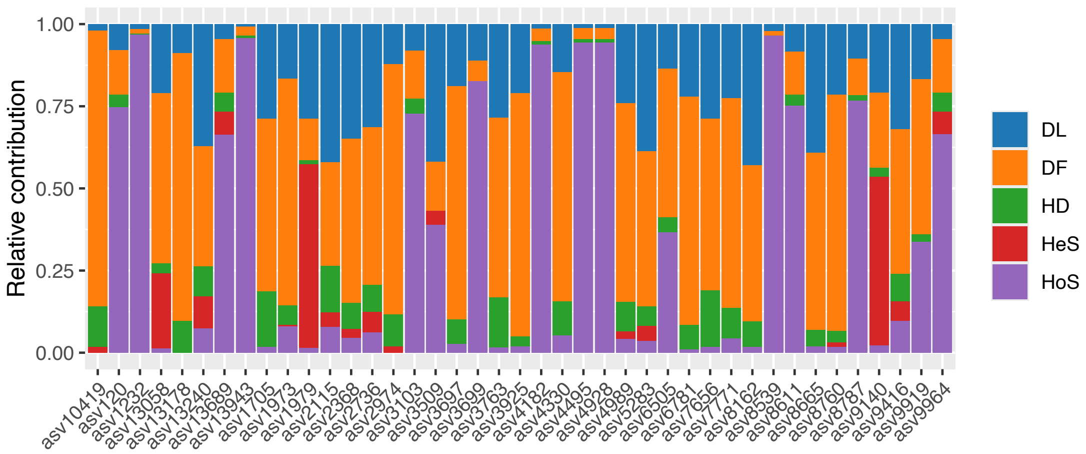

Figure S11. Relative contribution of ecological processes for the prokaryotic key species identified by co-occurrence network and random forest model. Five different ecological processes (homogeneous selection [HoS], heterogeneous selection [HeS], drift [DF], dispersal limitation [DL], and homogeneous dispersal [HD]) were analyzed.

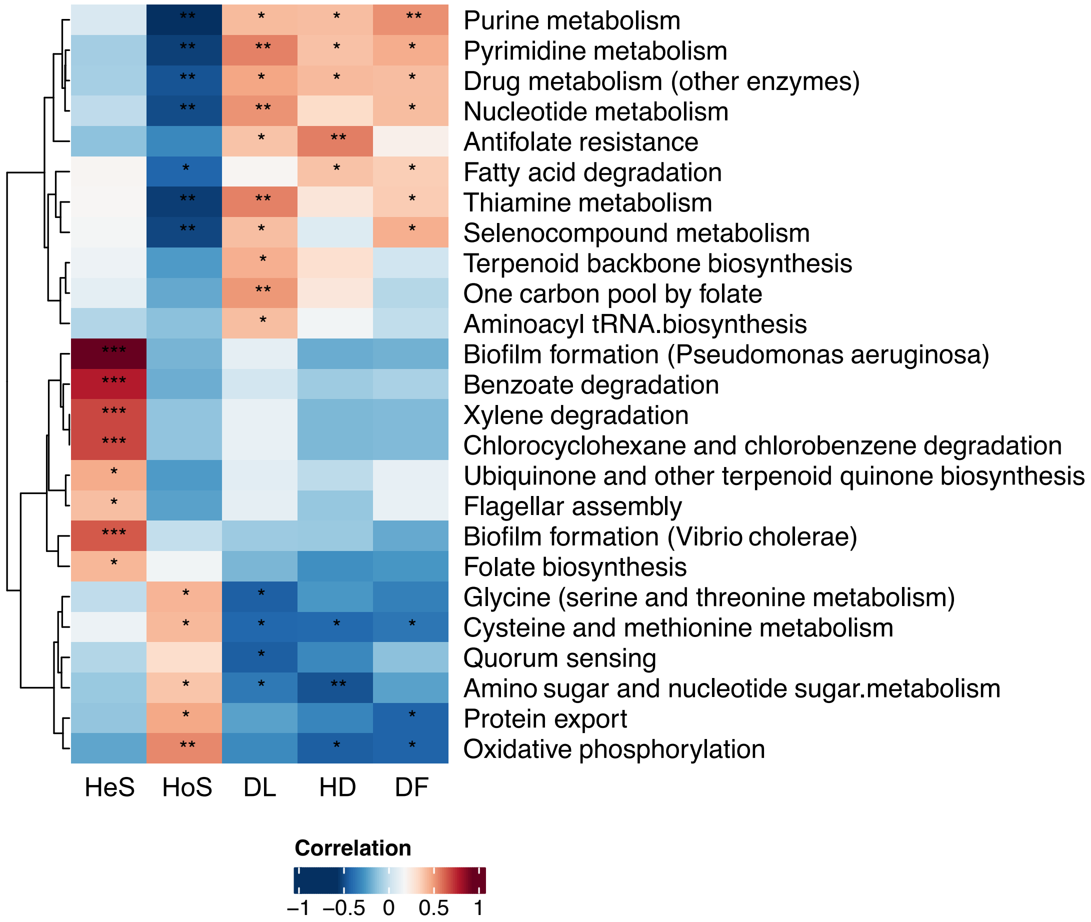

Figure S12 Pearson's correlation of metabolism enrichment of key species and ecological processes. The function of each key species was predicted using PICRUSt2. Five different ecological processes (homogeneous selection [HoS], heterogeneous selection [HeS], drift [DF], dispersal limitation [DL], and homogeneous dispersal [HD]) were analyzed. P values were adjusted using fdr method. *, adjusted p value < 0.05; **, adjusted p value < 0.01; ***, adjusted p value < 0.001. Only functions with a significant correlation to at least one ecological process are shown here.
